# Supplementary material for: Evidence for the formation of silicic lava by pyroclast sintering
Source: Nat Commun. 2024 Jun 24;15:5347. doi: 10.1038/s41467-024-49601-6 (PMC11196653; doi:10.1038/s41467-024-49601-6)
Supplement: Supplementary file 1 — Supplementary Information [file 41467_2024_49601_MOESM1_ESM.pdf]

Supplementary material 1

Photomicrographs of the upper and lower feeder dyke at Hrafntinnuhryggur, Iceland.
All scale bars are 0.5 mm.
Photomicrographs are oriented from dyke margin to margin so that up on the page is pointing towards the opposite margin (unless stated otherwise).
Sample number represents the distance away from the western margin (e.g., ‘sample 9’ is 9 cm away from the western margin).
Photomicrographs used in the manuscript have a red asterick and corresponding figure number in the bottom left hand corner.

Relict boundary - texture representing the joining of two of more clasts through sintering.
UD - Upper Dyke (see Figure 2k in the manuscript).
LD - Lower Dyke (see Figure 2l in the manuscript).

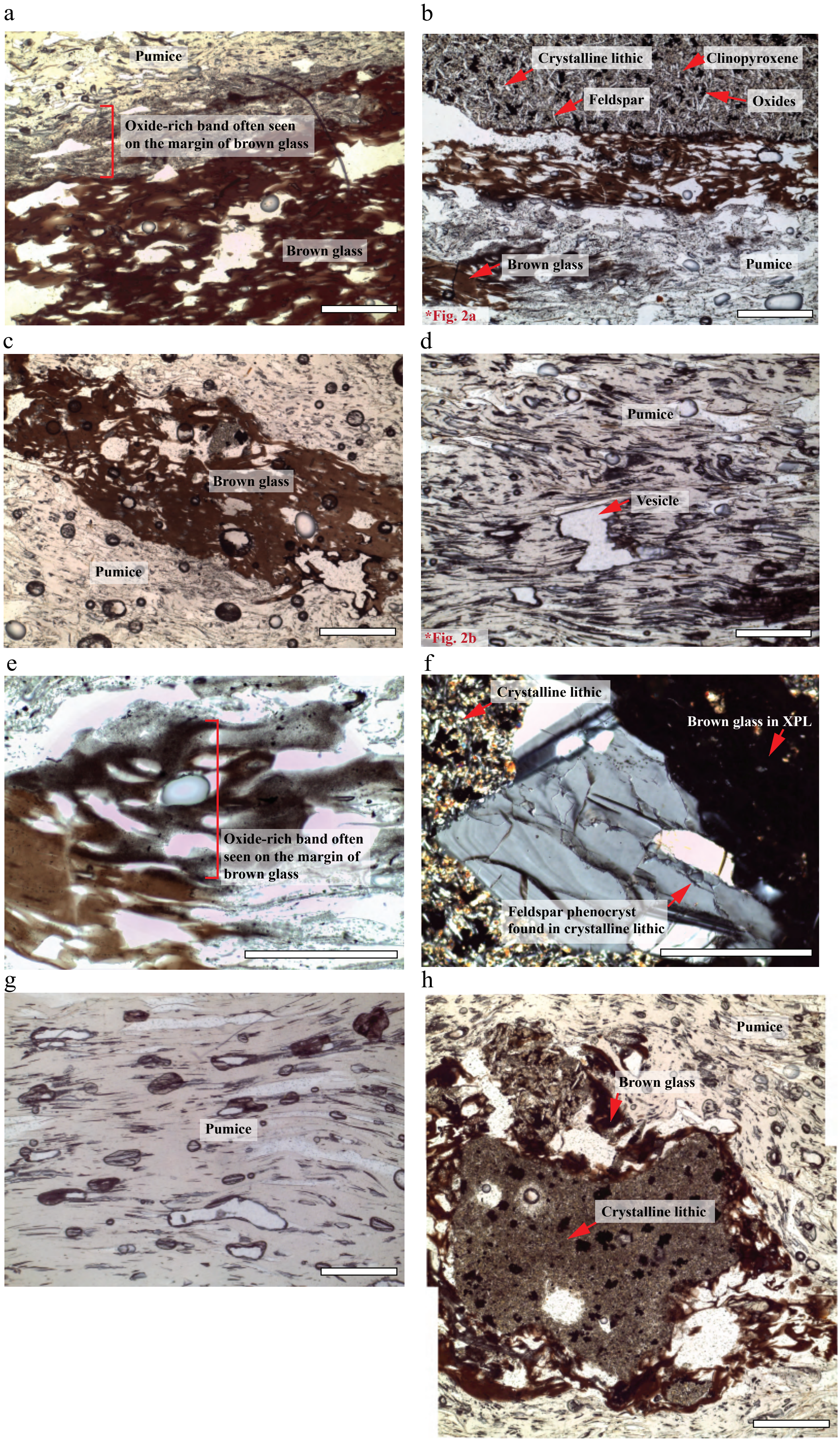

Supplementary Figure 1: Photomicrographs of the sample 0 from the lower dyke (LD). Sample 0 is a pumice lapilli tuff (pLT) which has bands of brown glass in the pumice groundmass. (a) Pumice with brown glass band. (b) Pumice with crystalline lithic and brown glass band. (c) Pumice with brown glass band. (d) Pumice with irregular vesicles. (e) Close-up on brown glass band seen in (a) and (c). (f) Crystalline lithic in pumice. (g) Pumice. (h) Crystalline lithic in pumice.

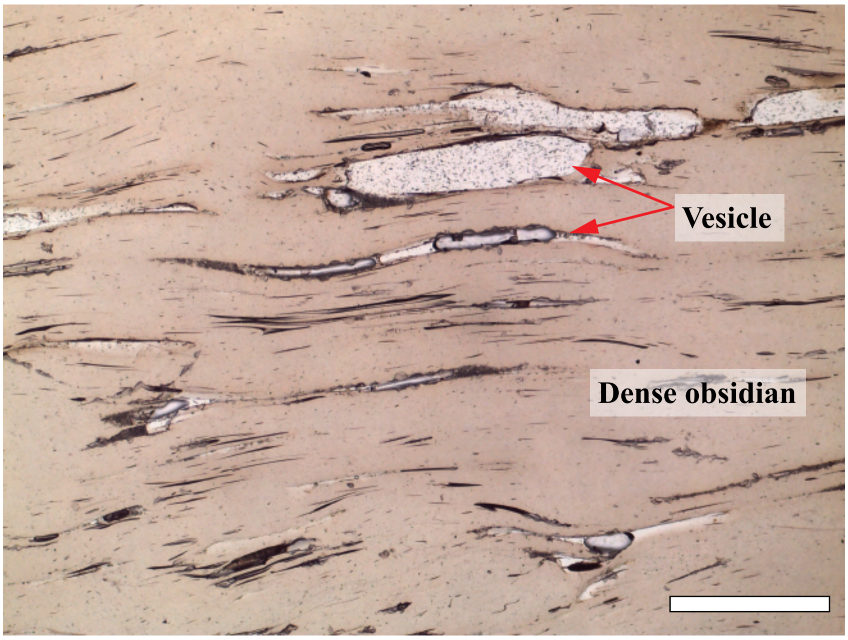

Supplementary Figure 2: Photomicrograph of sample LD 6. Sample 6 is a pumice which is welded near the western margin of the dyke.

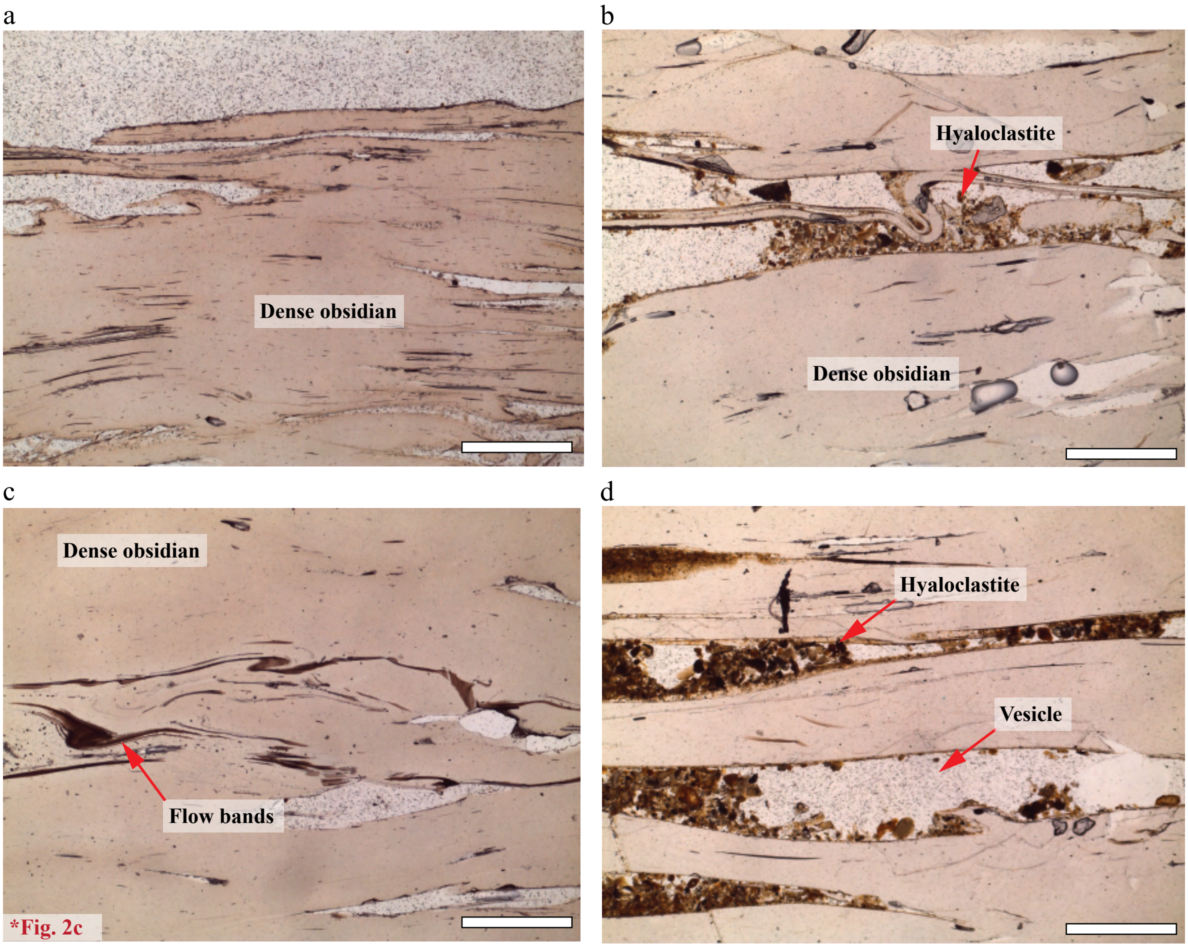

Supplementary Figure 3: Photomicrographs of sample LD 9. Sample 9 is a vesicular obsidian. (a) Obsidian with elongate vesicles. (b) Hyaloclastite lithics in a vesicle. (c) Brown flow bands in dense obsidian. (d) Hyaloclastite lithics in vesicles.

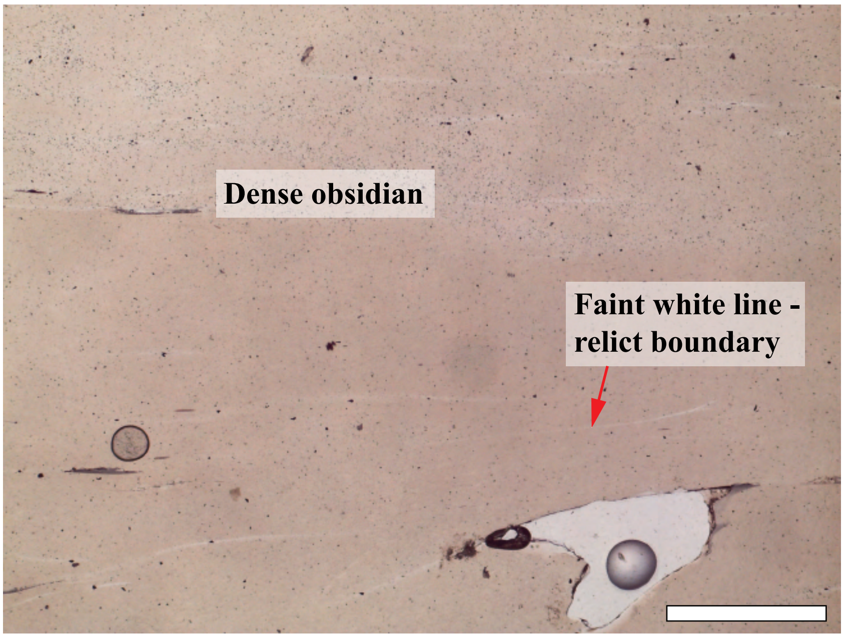

Supplementary Figure 4: Photomicrograph of sample LD 45. Sample 45 is a dense obsidian.

a

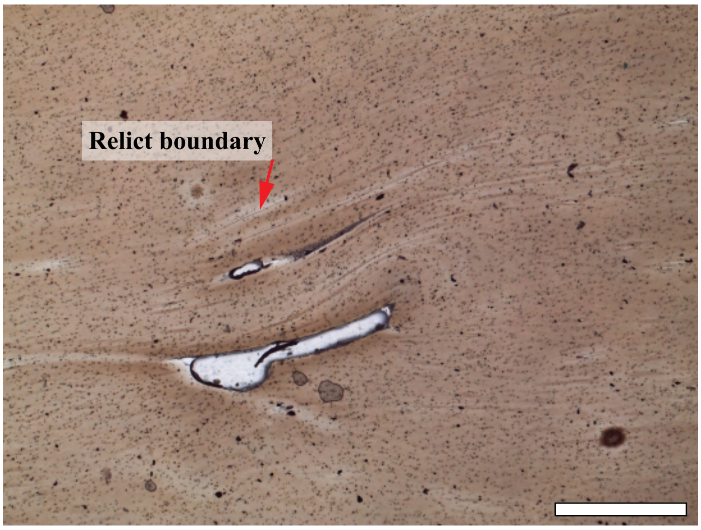

b

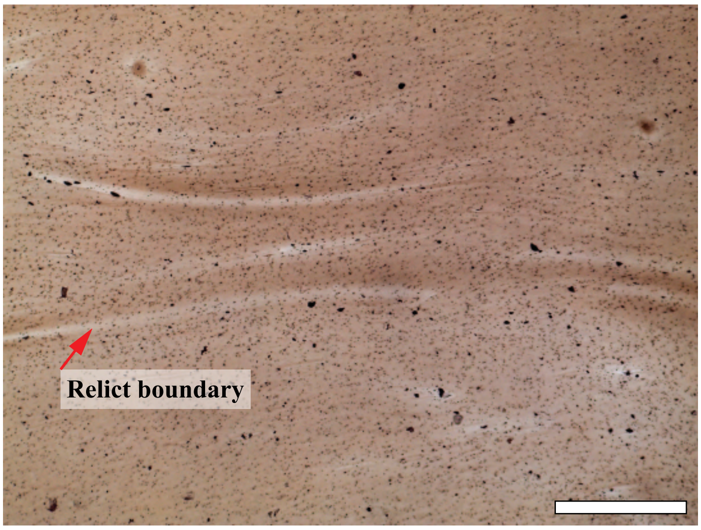

Supplementary Figure 5: Photomicrograph of sample LD 76. Sample 76 is a dense obsidian, with very few vesicles. (a) Dense obsidian with rare rounded vesicles. (b) Dense obsidian with faint white relict boundaries.

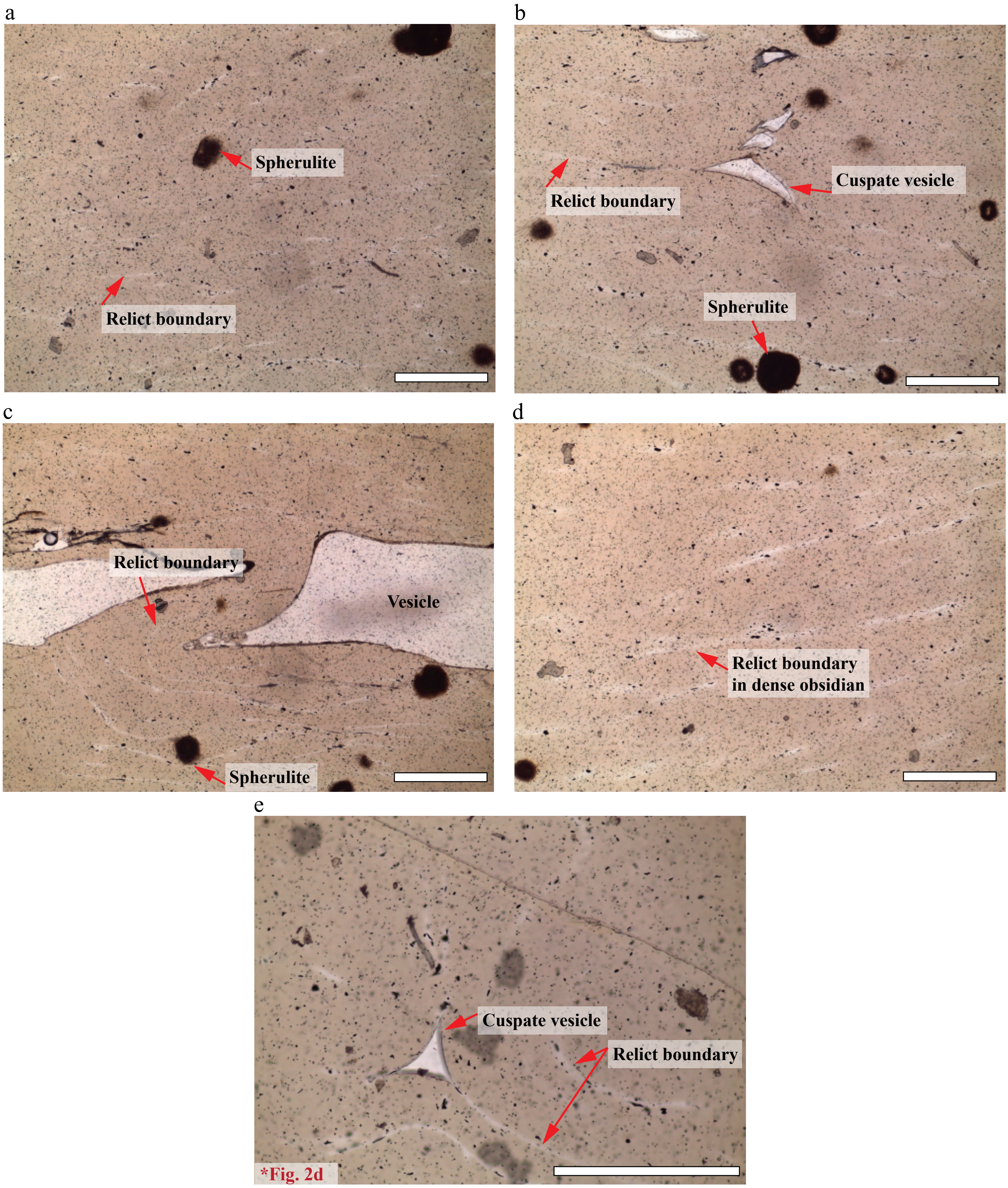

Supplementary Figure 6: Photomicrograph of sample LD 84. Sample 84 is a dense obsidian that has rare, irregular and cuspate vesicles. (a) Dense obsidian with spherulites and relict boundaries (faint white lines). (b) Dense obsidian with cuspate vesicles. (c) Dense obsidian with irregular vesicles. (d) Dense obsidian. (e) Dense obsidian with a tri-cuspate vesicle.

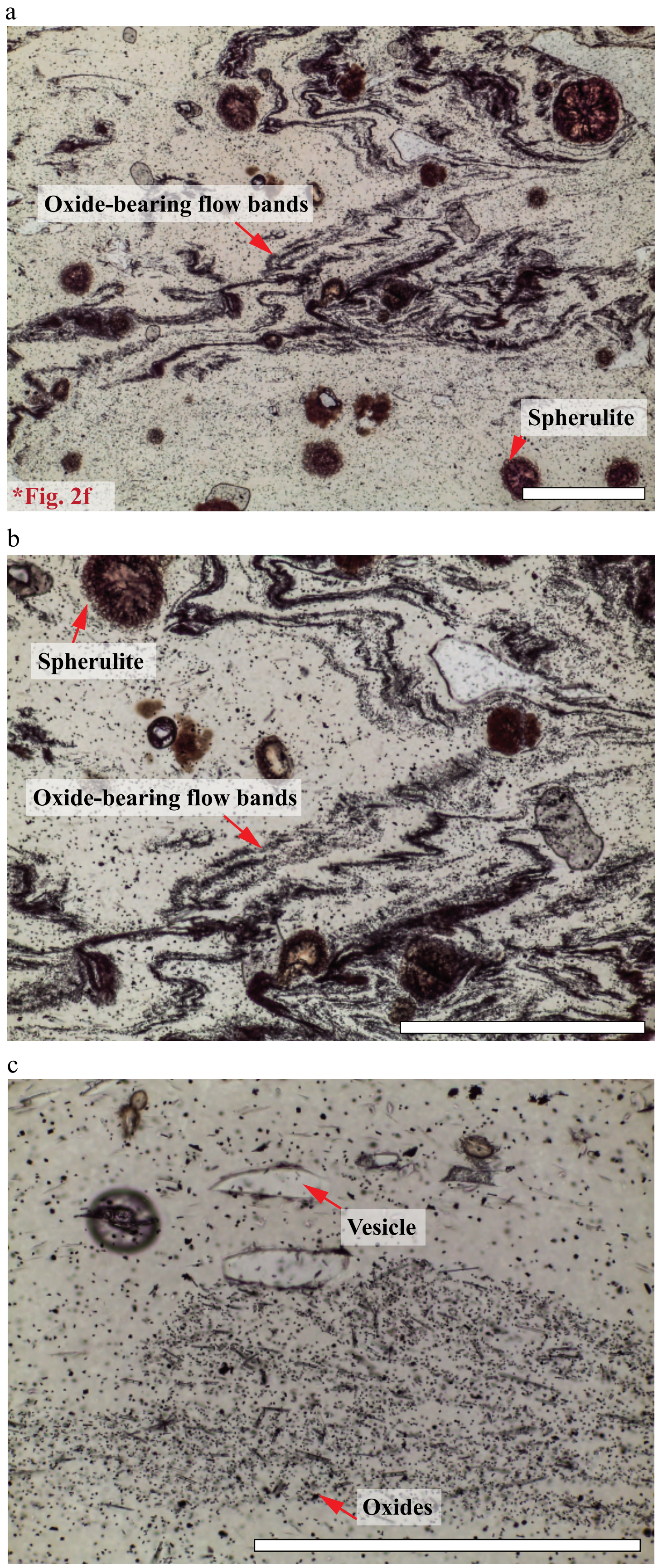

Supplementary Figure 7: Photomicrographs of the sample LD 92. Sample 92 contains abundant microlites and spherulites. (a & b) Obsidian with oxide flow bands. (c) Obsidian with oxides and small subrounded vesicles.

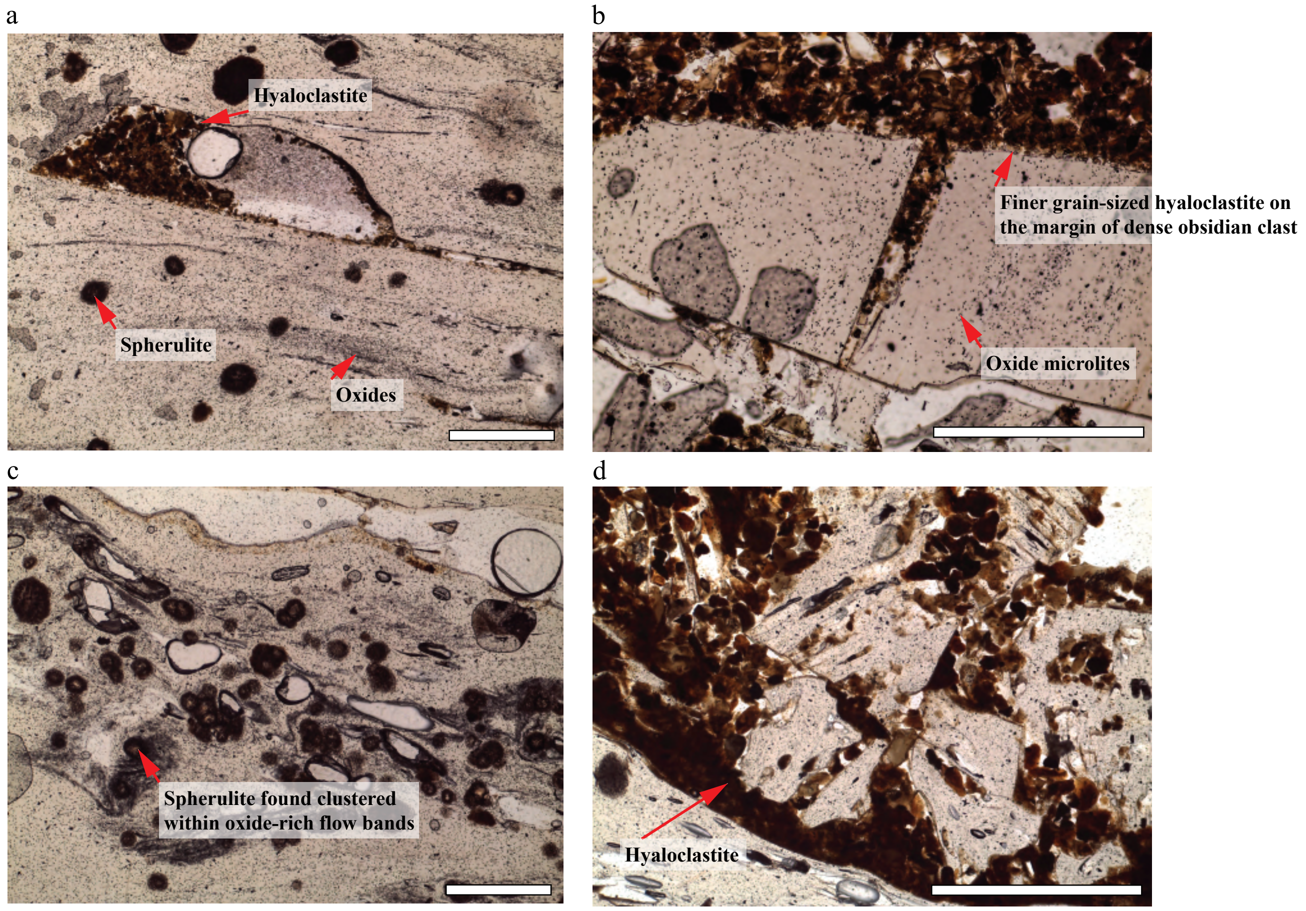

Supplementary Figure 8: Photomicrographs of the sample LD 93. Sample 93 contains abundant microlites and spherulites. (a) Hyaloclastite in an enclosed vesicle. (b) Zoomed in of the hyaloclastite in the a vesicle. (c) Abundant spherulites in oxide-rich obsidian. (d) Broken obsidian clasts and hyaloclastite in a vesicle.

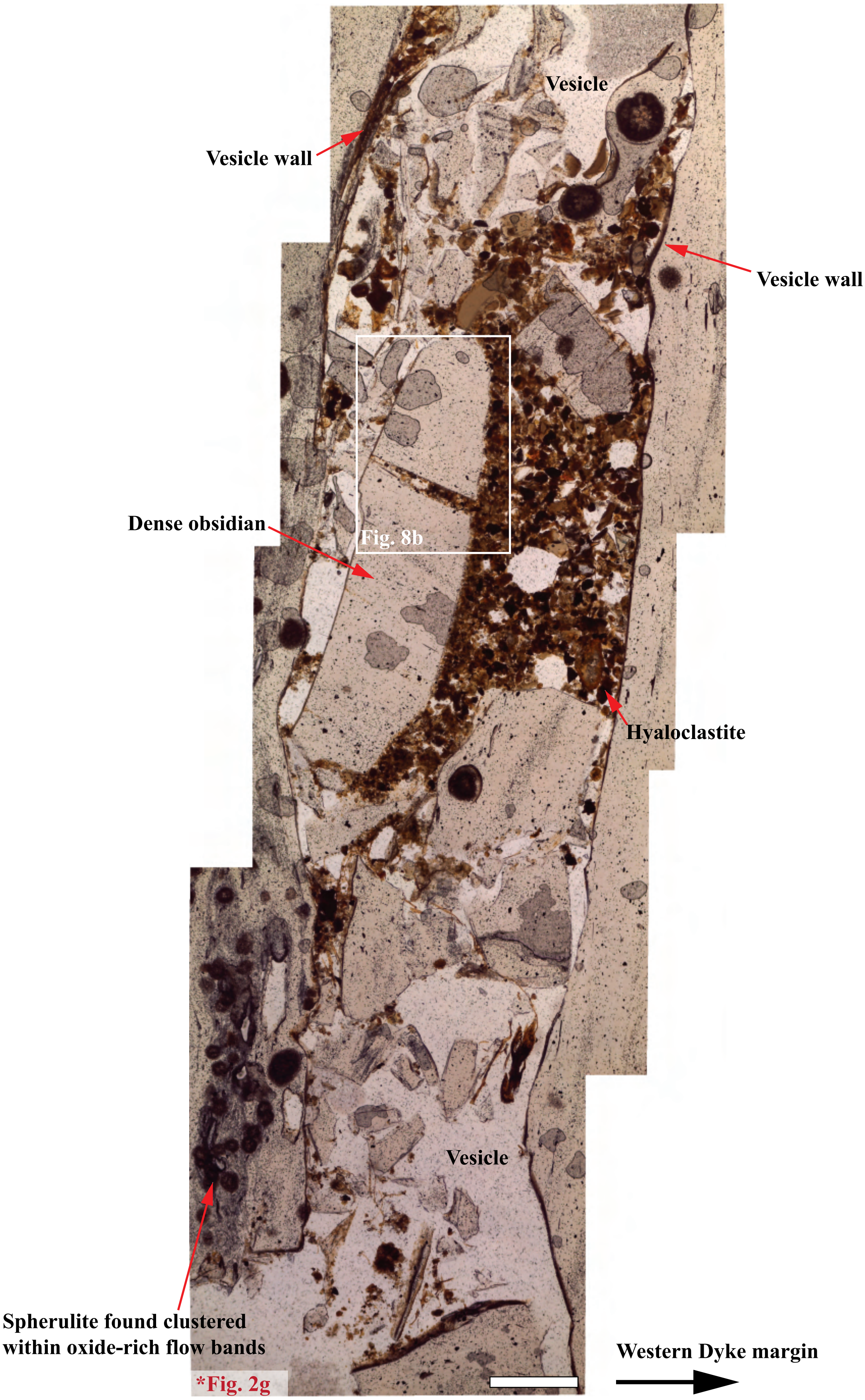

Supplementary Figure 9: Photomicrograph mosaic of sample LD 93.

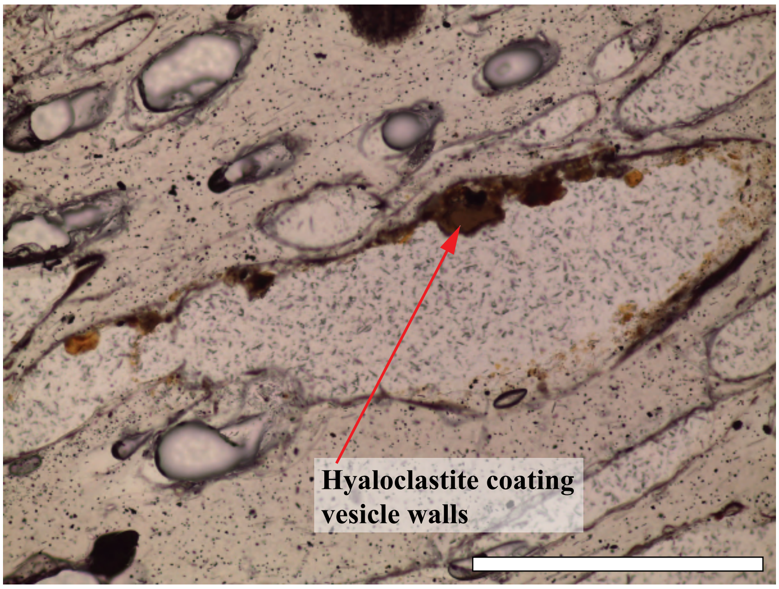

Supplementary Figure 10: Photomicrograph of sample LD 101. Sample 101 is a vesicular obsidian with hyaloclastite lithics in vesicles.

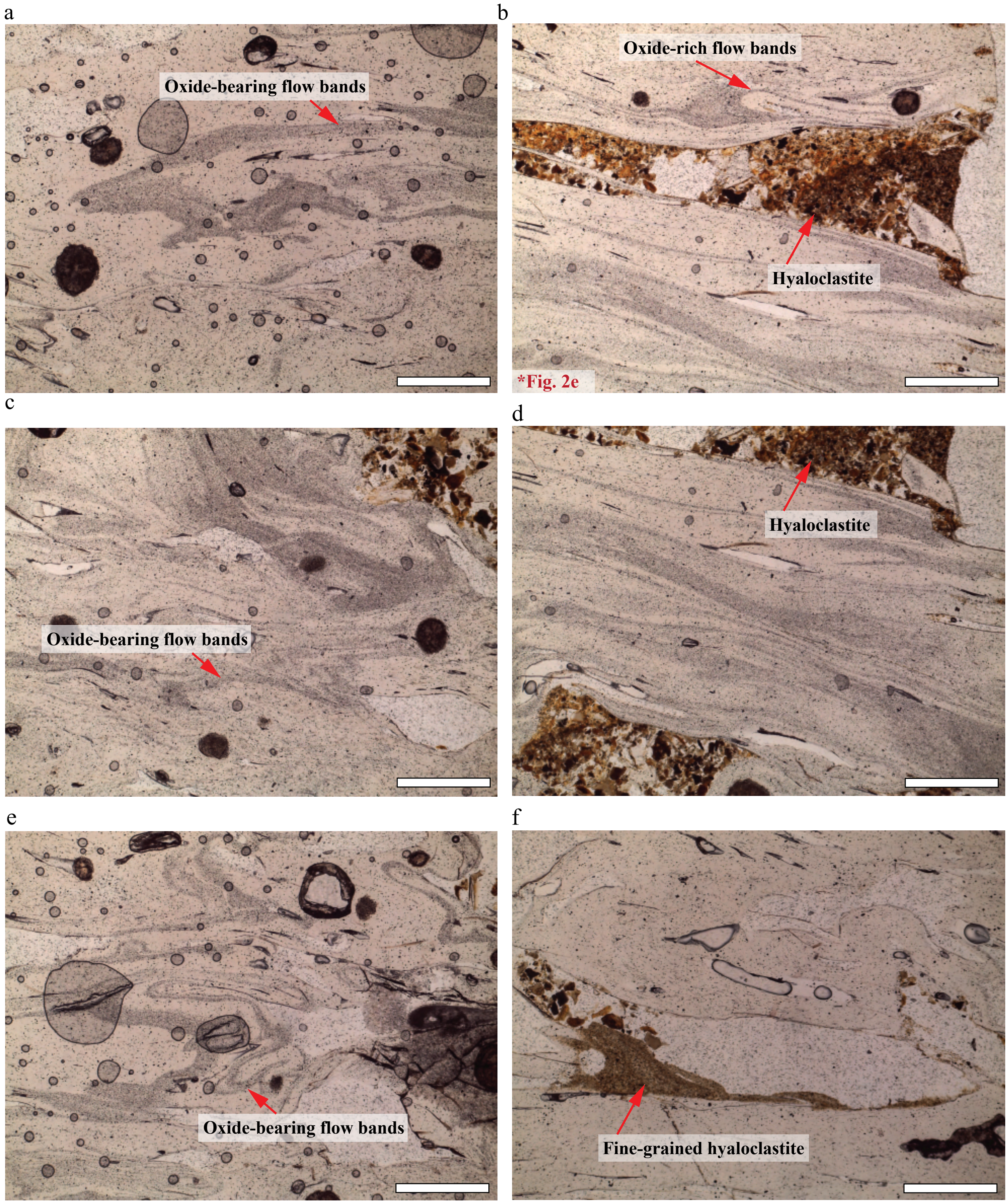

Supplementary Figure 11: Photomicrograph of sample LD 110. Sample 110 is a microlite-bearing obsidian and has microlite flow-banding. Hyaloclastite lithics are often found in vesicles in this sample. (a) Obsidian with oxide flow bands. (b, c & d) Obsidian with oxide flow bands and hyaloclastite lithics in vesicles. (e) Obsidian with oxide flow bands. (f) Obsidian with fine-grained hyaloclastite lithics in a vesicle.

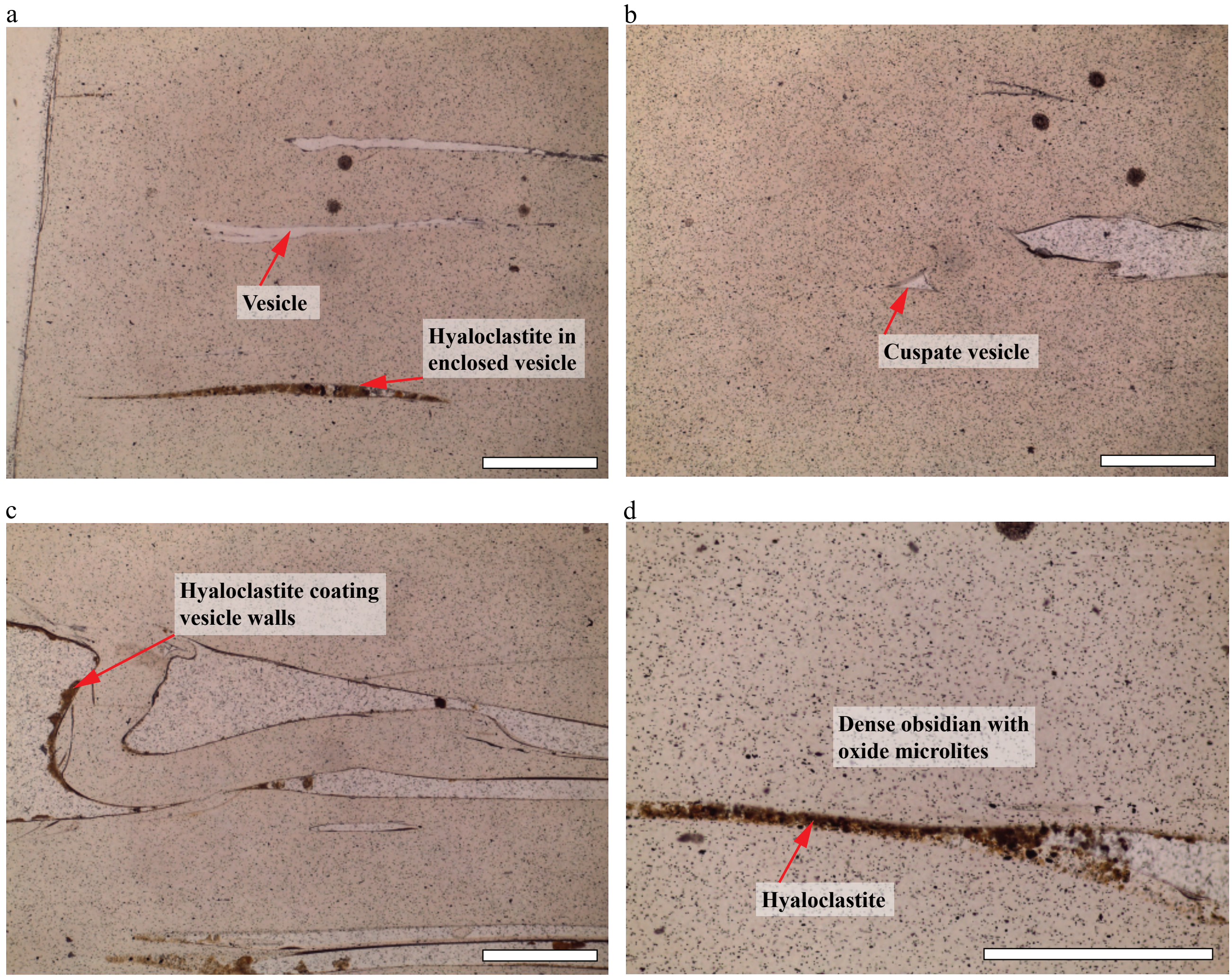

Supplementary Figure 12: Photomicrographs of the sample LD 125. Sample 125 is a microlite-bearing obsidian which often has hyaloclastite lithics in vesicles. (a) Obsidian with elongate vesicles and hyaloclastite lithics in an elonagate vesicle. (b) Cuspate vesicle in obsidian. (c) Obsidian with hyaloclastite coating vesicle walls. (d) Close-up of hyaloclastite in a vesicle.

a

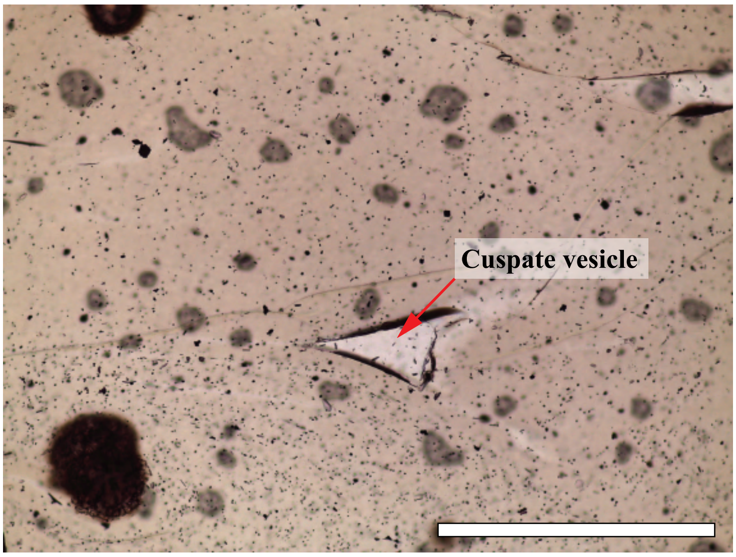

b

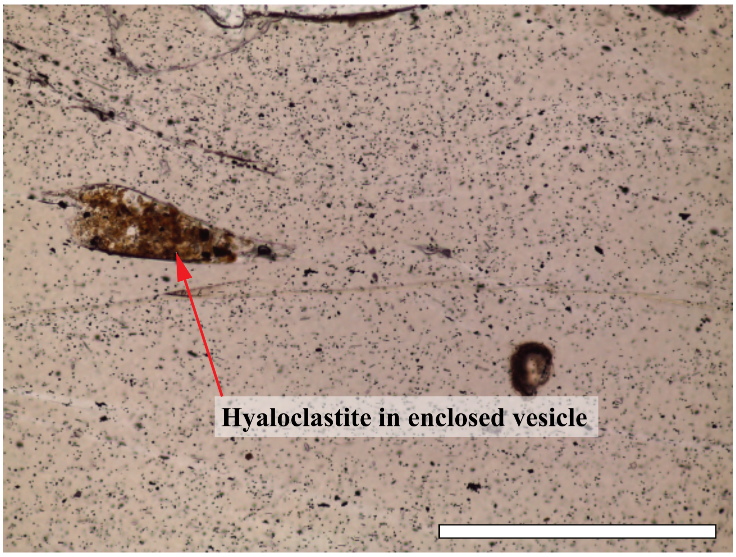

Supplementary Figure 13: Photomicrographs of the sample LD 140. Sample 140 is a microlite-bearing obsidian. (a) Cuspate vesicle in obsidian. (b) Hyaloclastite in a vesicle.

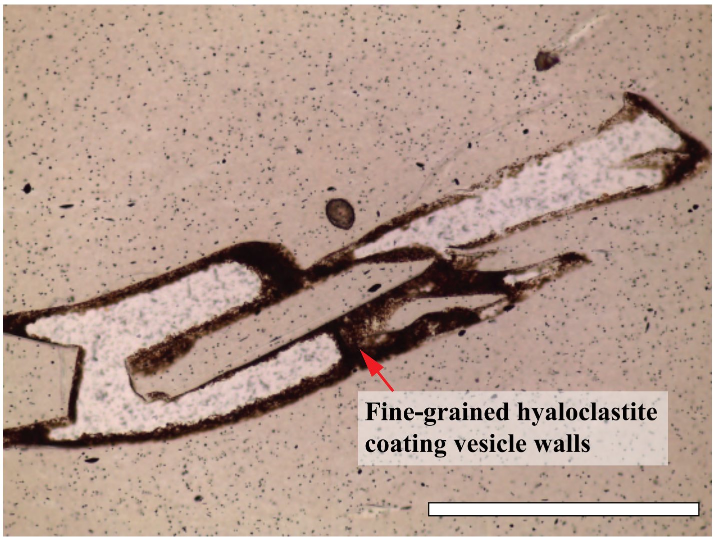

Supplementary Figure 14: Photomicrograph of the sample LD 193.

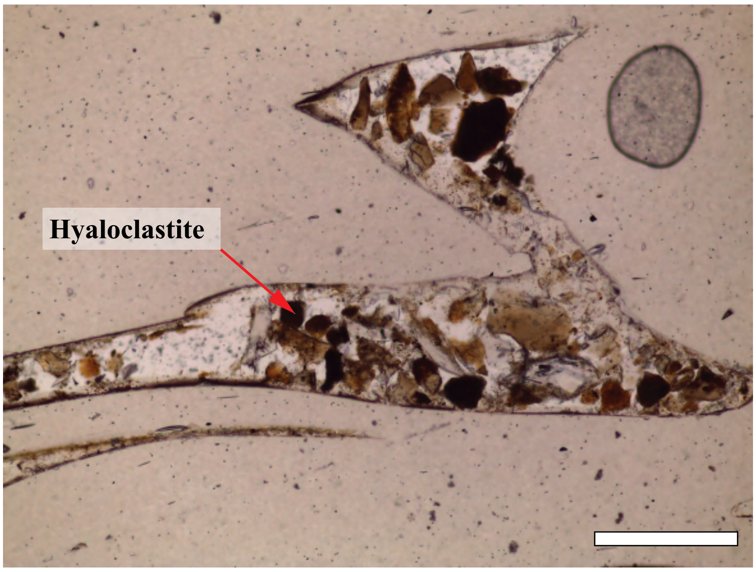

Supplementary Figure 15: Photomicrograph of sample LD 222.

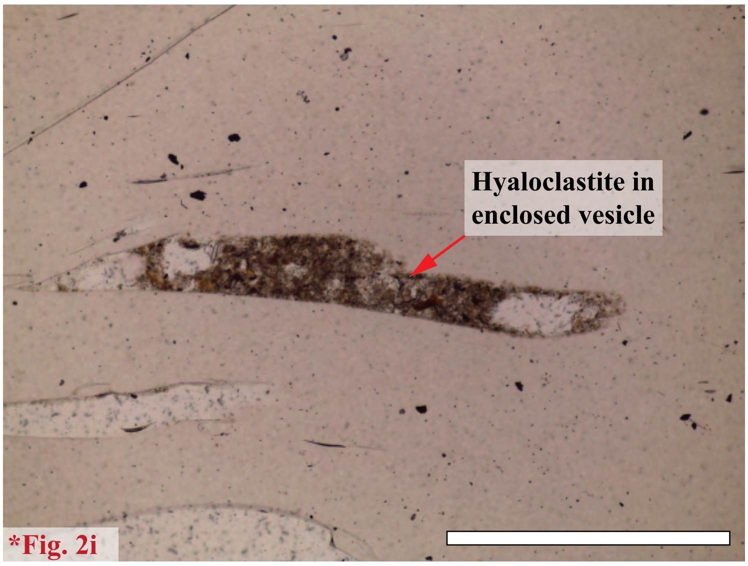

Supplementary Figure 16: Photomicrograph of sample LD 234.

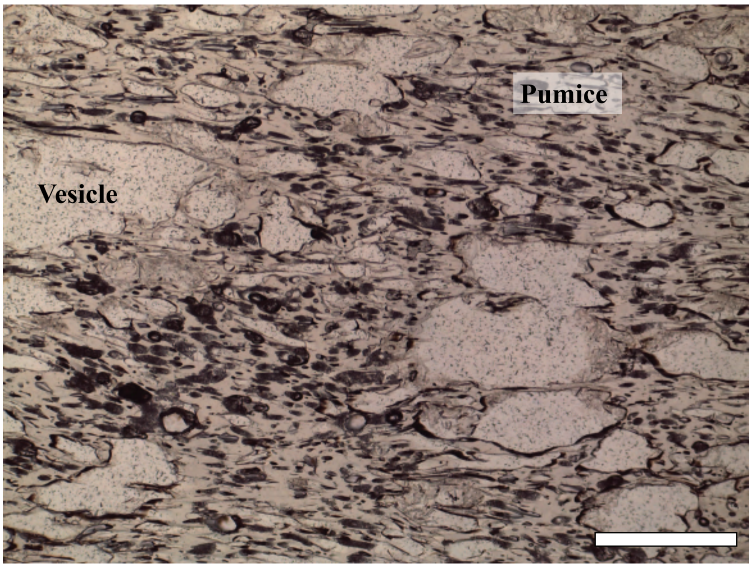

Supplementary Figure 17: Photomicrograph of sample 1 from the upper dyke (UD). Sample number represents the distance in cm from the western margin of the dyke. Sample 1 is a pumice lapilli tuff (pLT).

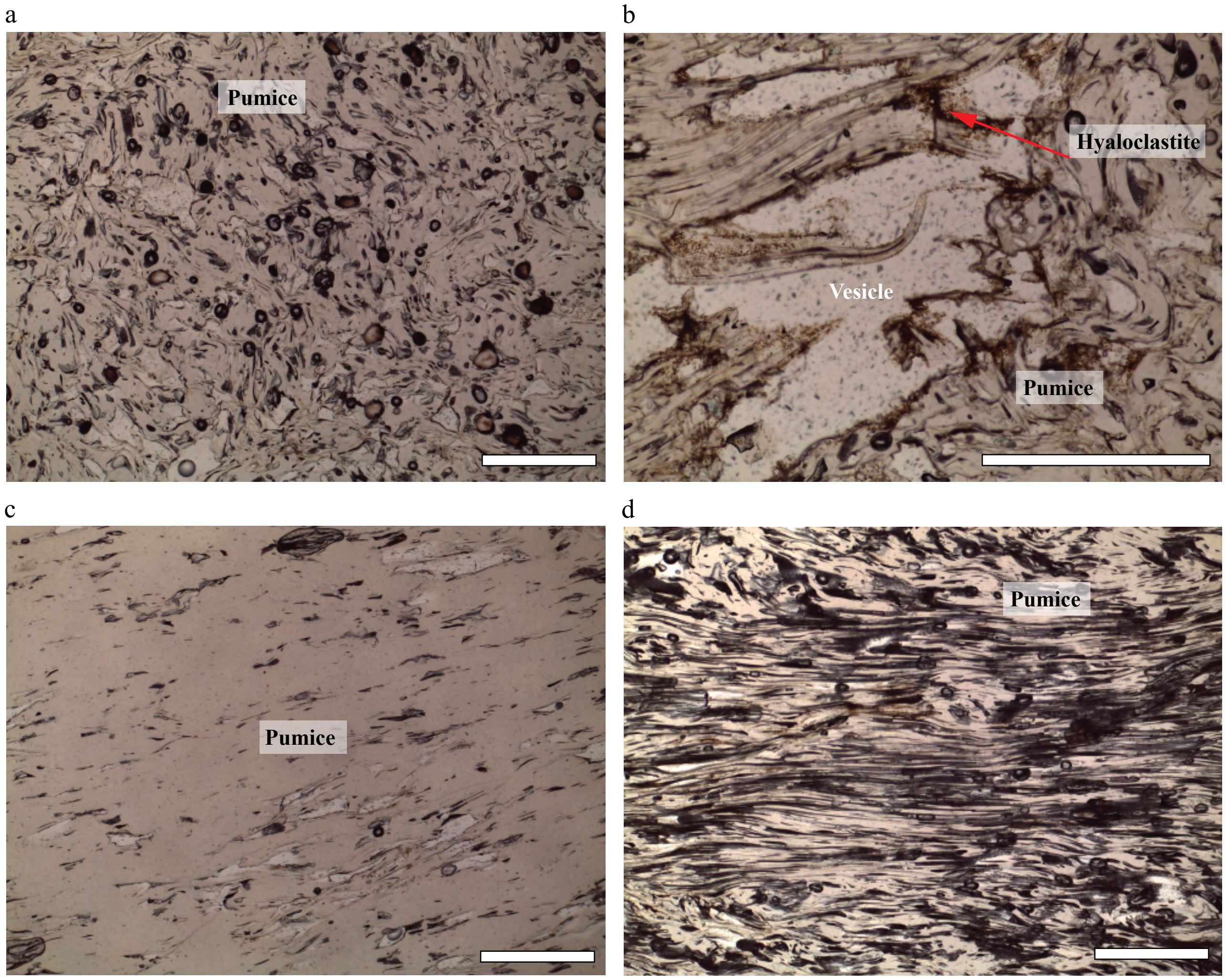

Supplementary Figure 18: Photomicrographs of sample UD 12. (a) Pumice. (b) Pumice with hyaloclastite in vesicles. (c & d) Pumice.

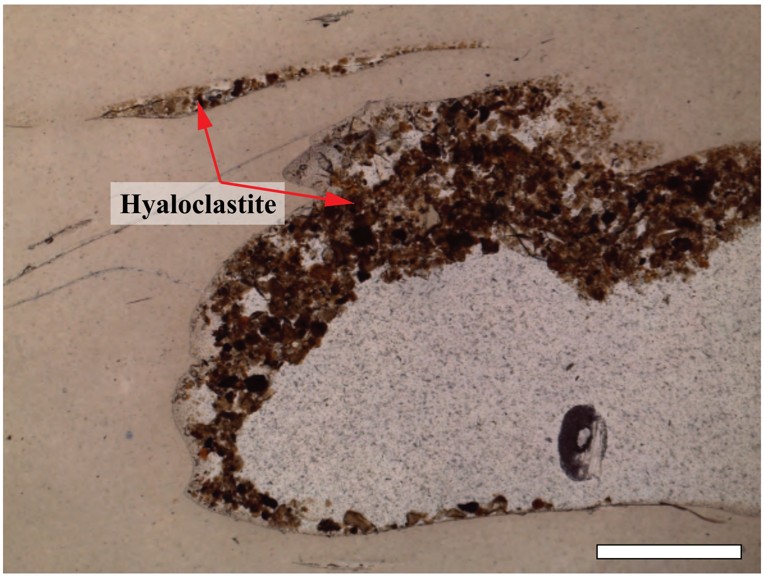

Supplementary Figure 19: Photomicrograph of sample UD 15.

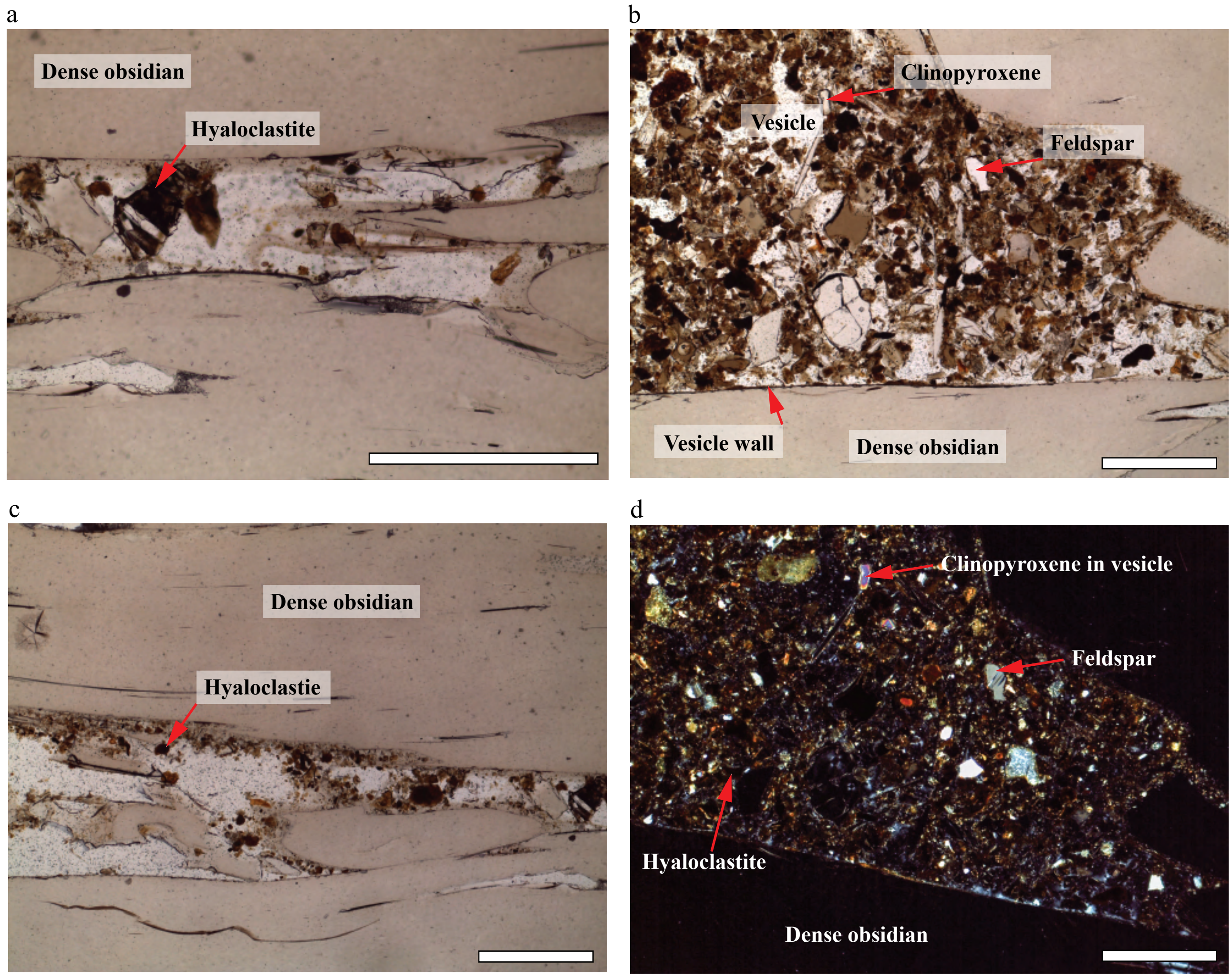

Supplementary Figure 20: Photomicrographs of sample UD 20. Sample 20 is an obsidian. (a, b & c) Dense obsidian with vesicles containing hyaloclastite lithics. (d) Cross polarised light photomicrograph of (b).

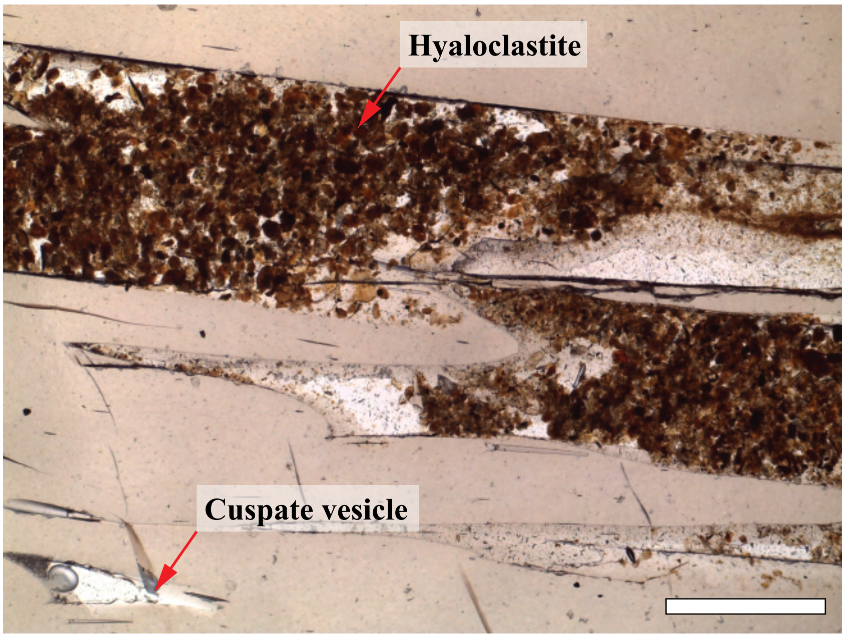

Supplementary Figure 21: Photomicrograph of the sample UD 32.

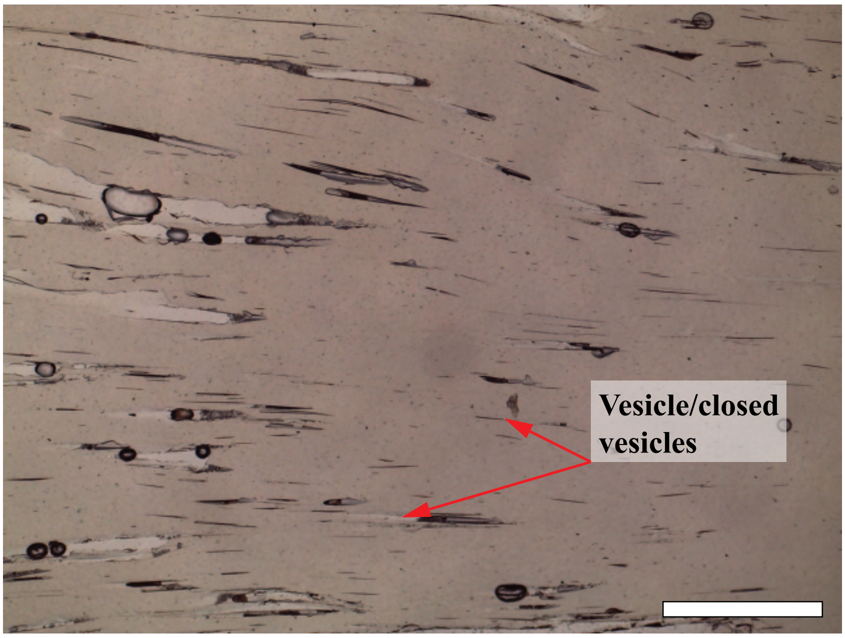

Supplementary Figure 21: Photomicrograph of the sample UD 35.

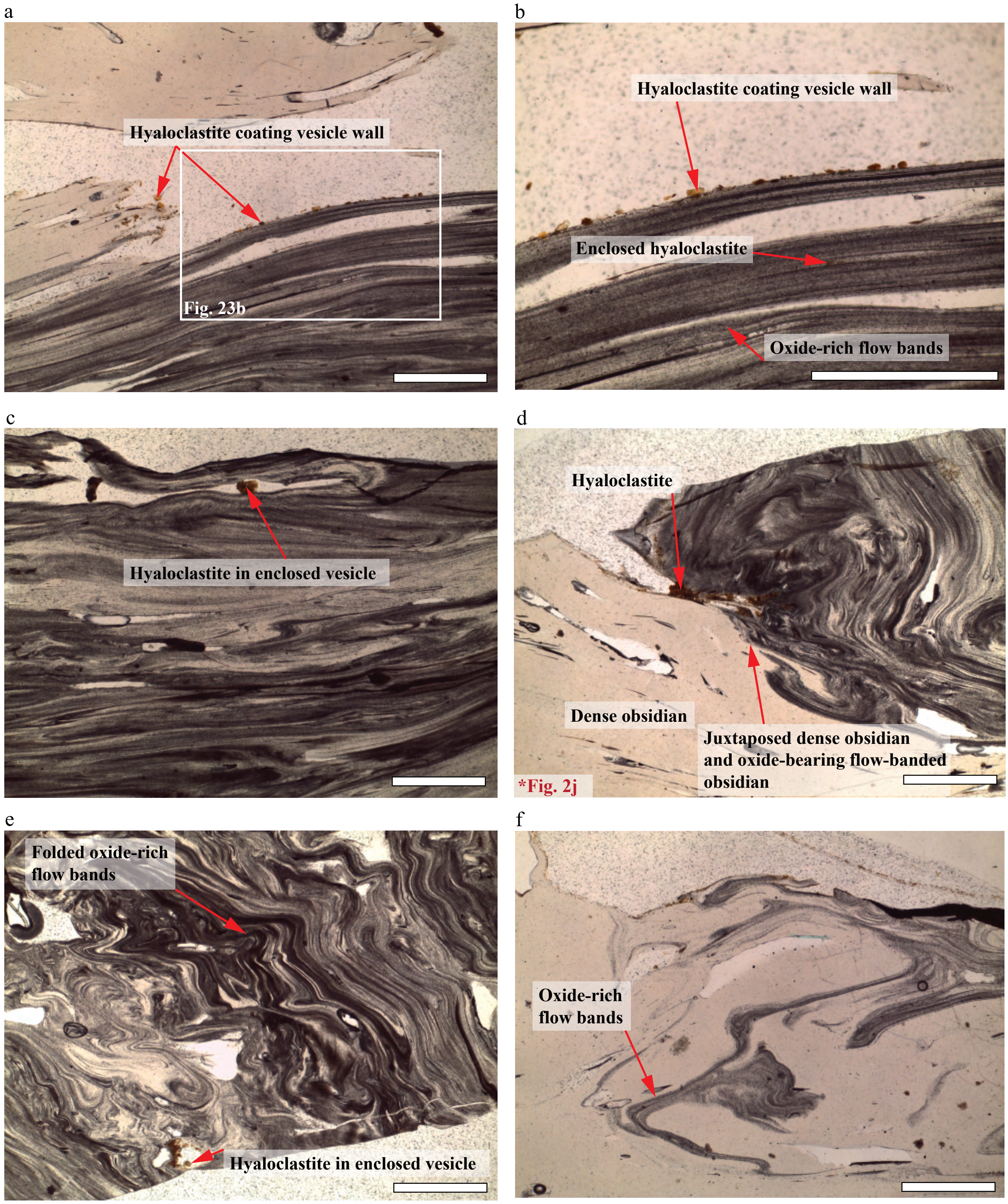

Supplementary Figure 23: Photomicrographs of sample UD 50. Sample 50 is a dense obsidian which has folded oxide-rich flow bands. (a) Flow banded obsidian juxtaposed with featureless obsidian. Hyaloclastite lithics are found on vesicle walls. (b) Close-up of (a). (c) Flow banded obsidian with hyaloclastite lithics in enclosed vesicle. (d) Flow banded obsidian juxtaposed with featureless obsidian. (e) Folded flow bands. (f) Oxide-rich flow bands in obsidian.

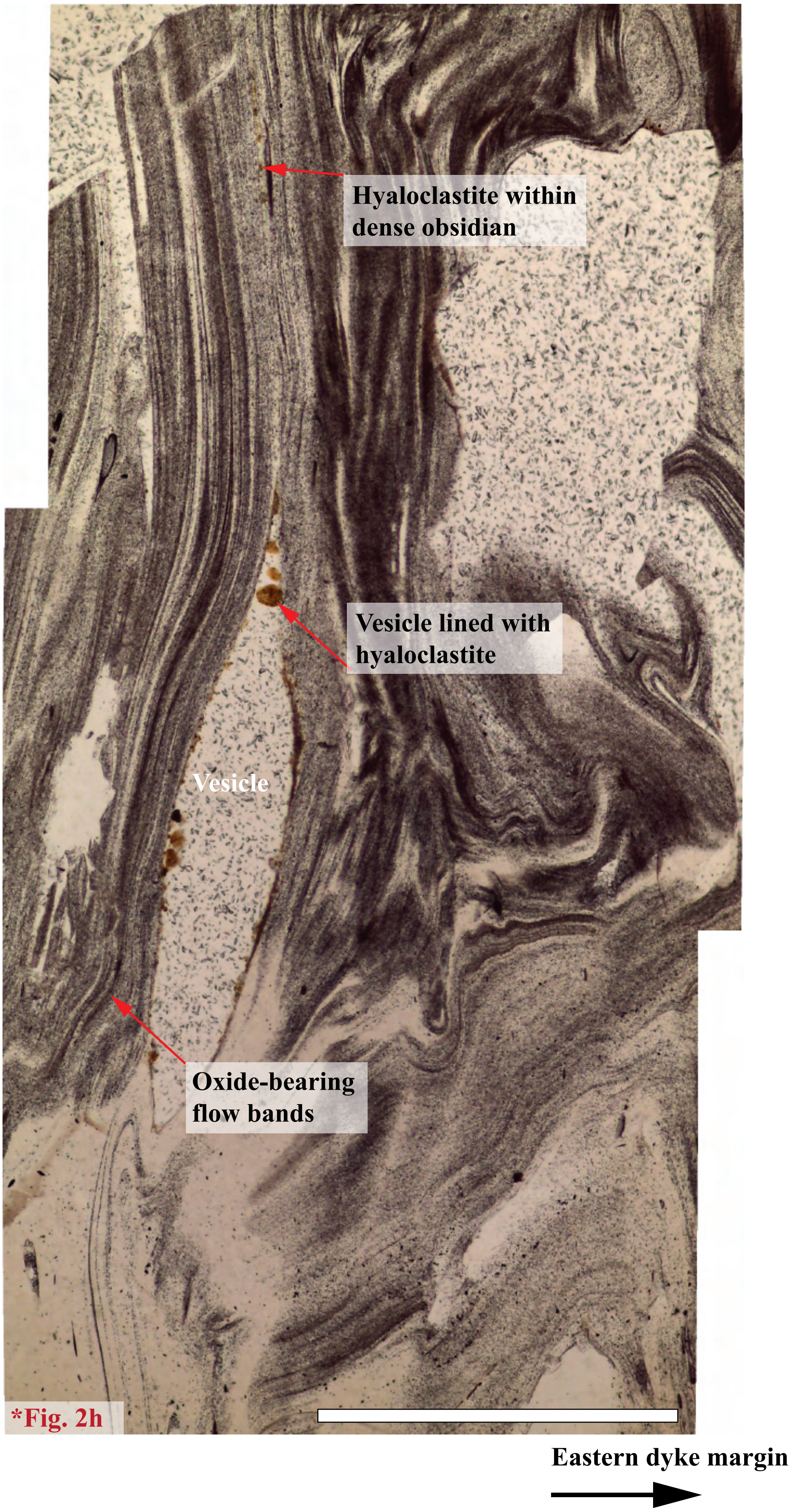

Supplementary Figure 24: Photomicrograph of the sample UD 50.

Supplementary material 2

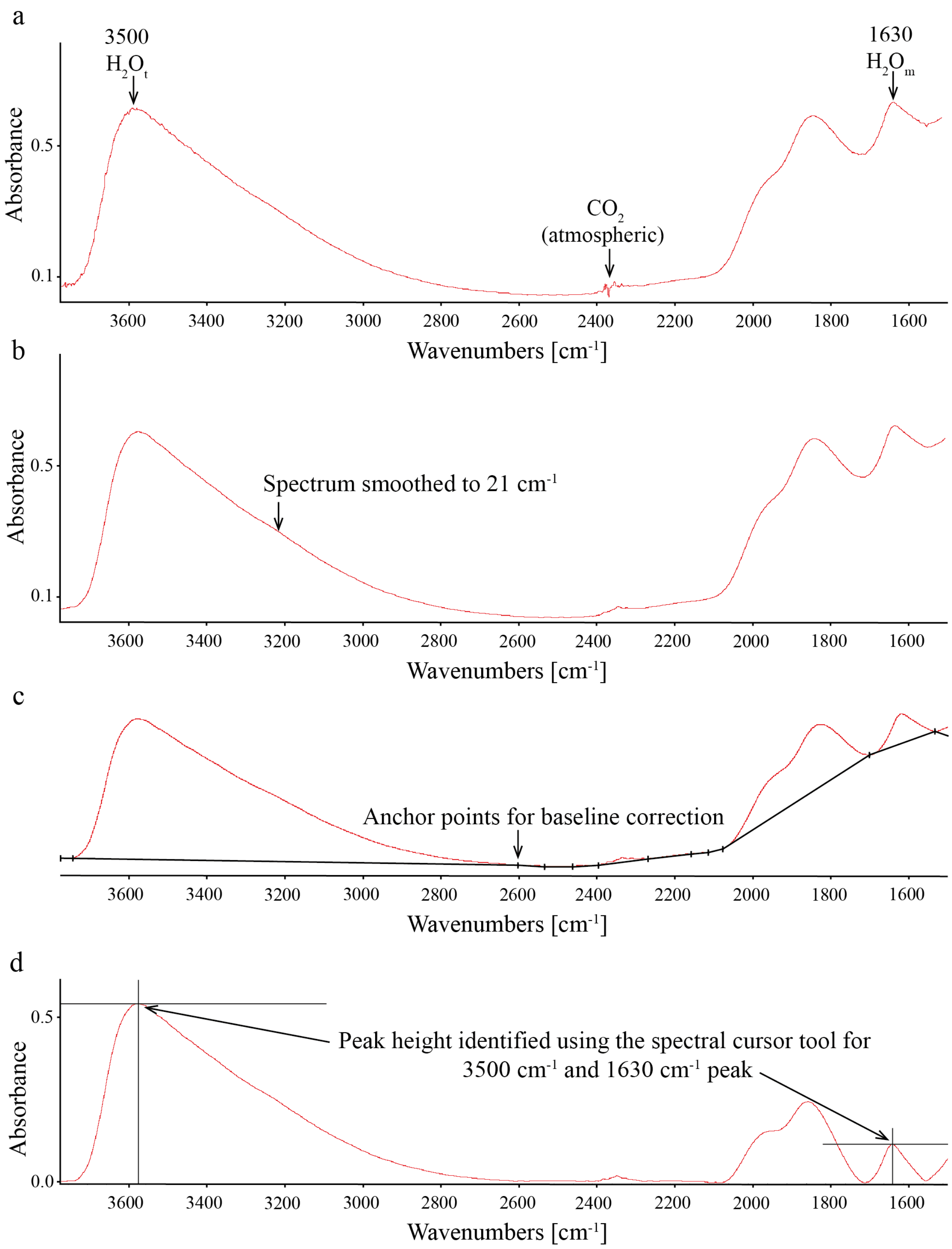

Supplementary Figure 25: Example of the processing workflow for an individual spectrum using Thermo Scientific software OMNIC. (a) Raw spectrum. (b) Spectrum after it has undergone smoothing. (c) Black line is the manually added baseline and the arrow indicated anchor points. (d) Peak height is identified using the spectral cursor tool and the absorbance value is noted next to the sample and spectrum number. See FTIR data.xlsx in the supplementary material.

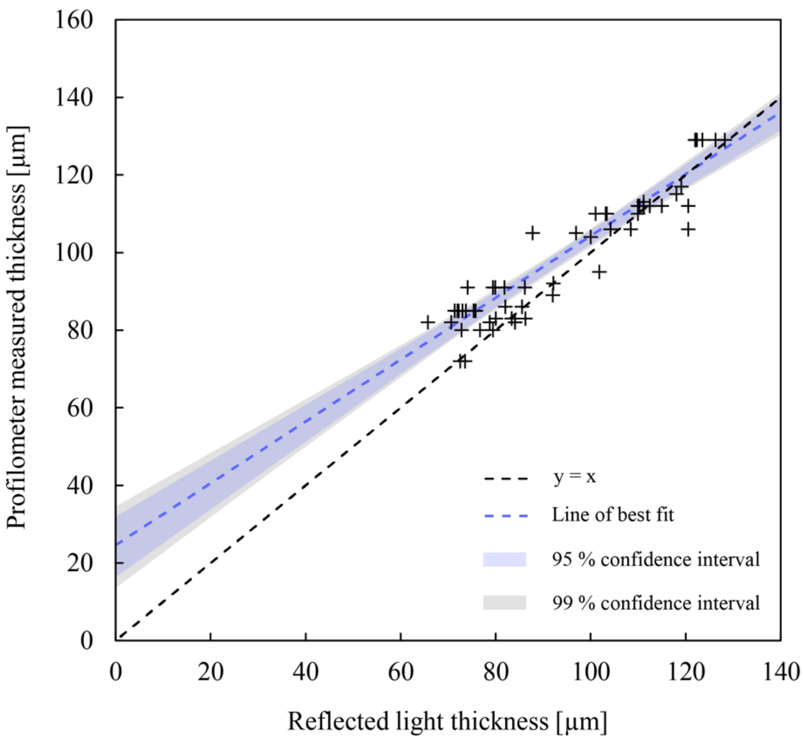

Supplementary Figure 26: Comparison of wafer thickness measured from a profilometer and reflected light mode on FTIR. For data please see FTIR wafer thickness.xlxs in the supplementary material.

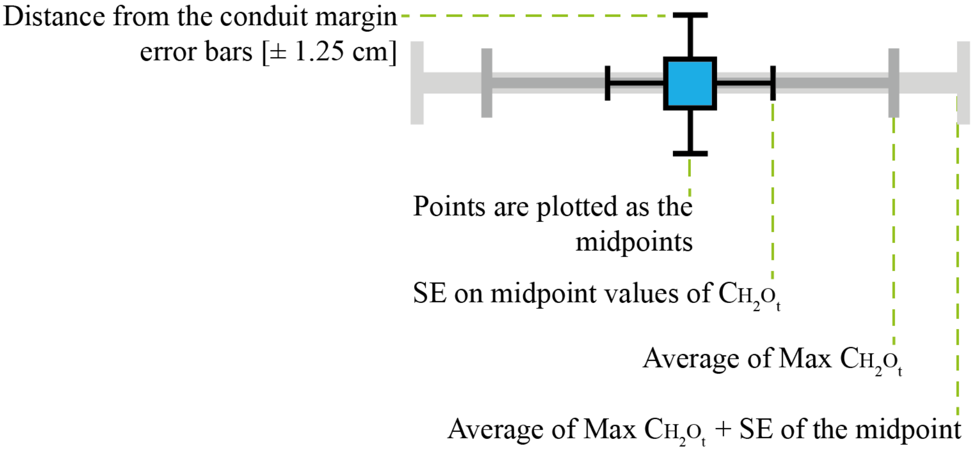

Supplementary Figure 27: x-axis and y-axis error bars as seen on Fig. 2o and p. The x-axis error bars are for total water concentration (H_2O_t wt %) and they represent; (1) black error bars are the standard error on midpoint values for H_2O_t wt %, (2) darker grey error bars are the average of the sum of the minimum H_2O_t values, (3) pale grey and the largest error bars are both the black and the darker grey error bars combined. The y-axis error bar is for distance from the feeder dyke margin has an error bar of 1.25 cm as the average sample size was approximately 3 cm.

Supplementary material 3

Mosaic photomicrograph maps of Hrafntinnuhryggur obsidian wafers produced on the FTIR. Maps show data spots that were collected for either transmitted (water spectra) or reflected (wafer thickness) light.
Sample number represents the distance away from the western margin (e.g. UD 9 is 9 cm away from the western margin on the upper dyke transect). Spectra and reflected mode map number are reported in FTIR.xlsx. Some spectra were disregarded as they were too noisy.
All scale bars are 1 mm (unless stated otherwise).

Green crosses (+) are background measurements used for transmitted light spectra.
Blue crosses (+) mark where measurements were collected in transmitted light for water data.
Red crosses (+) mark either where data was collected for wafer thickness in reflected light mode or a selected spectra spot on the transmitted light maps.
Note that some reflected light mode maps have blue crosses instead of red as they were immediately re-ran after transmitted light mode.

pLT - pumice lapilli tuff.

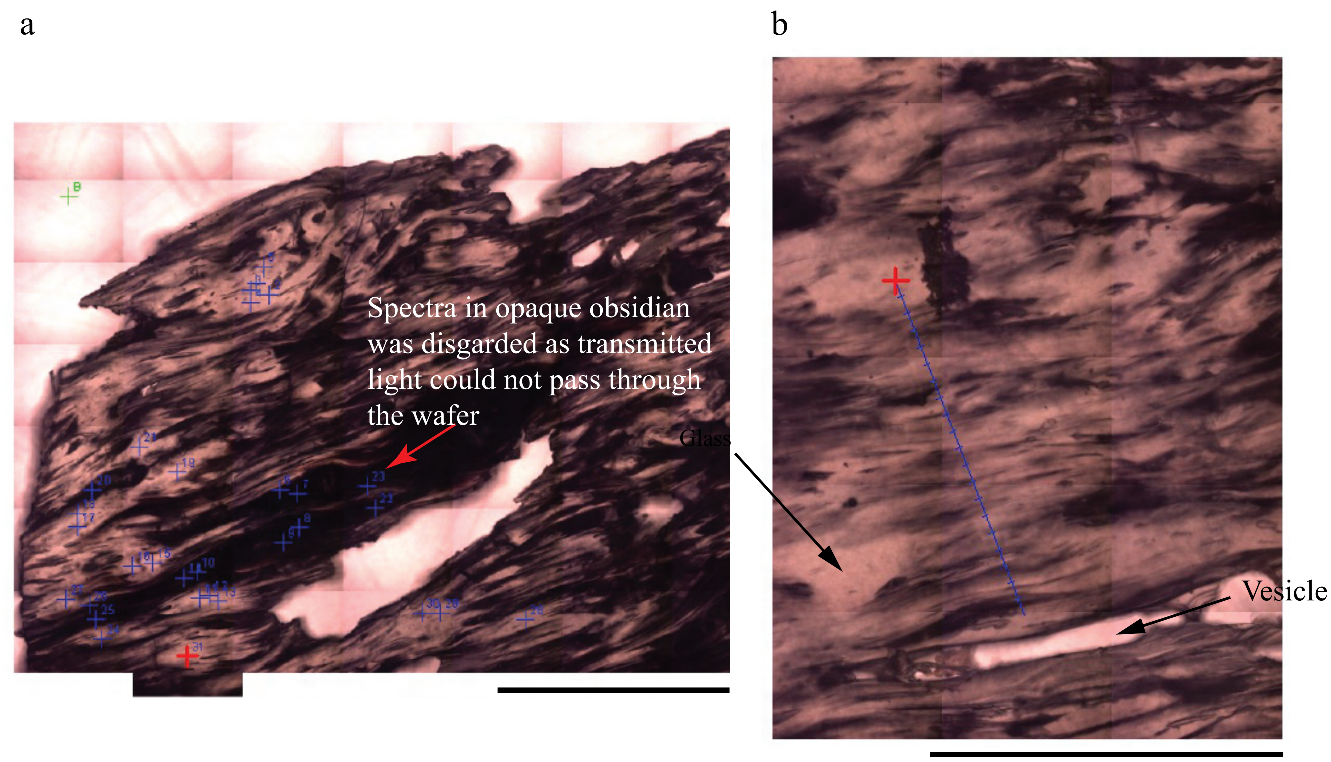

Supplementary Figure 28: (a) Transimitted light mode spectra map of pumice lapilli tuff (pLT) from the lower dyke (LD), labelled as Map1 in FTIR data.xlsx. (b) Transimitted light mode line map of pLT from LD labelled as Line1 in FTIR data.xlsx.

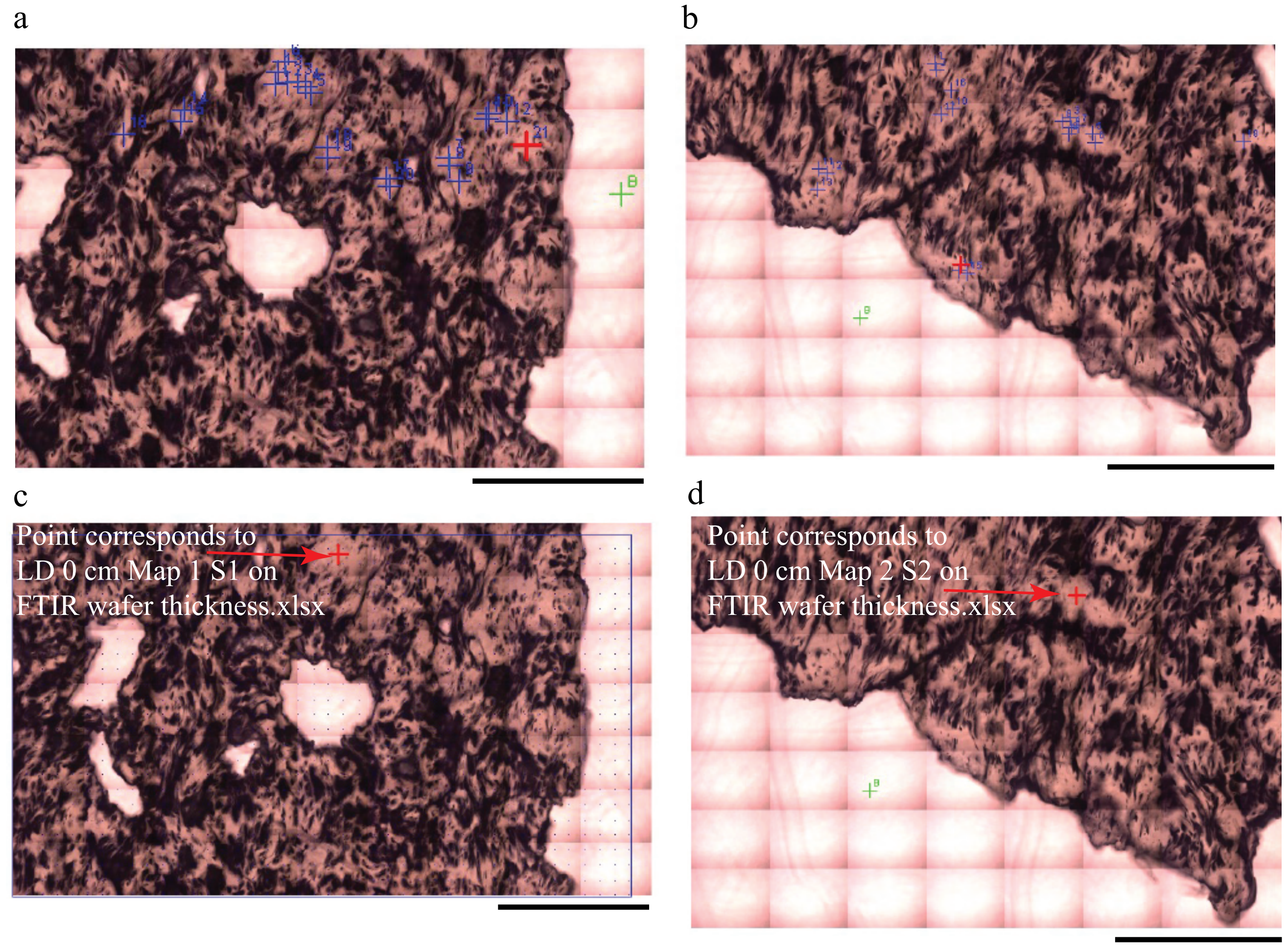

Supplementary Figure 29: (a) Transimitted light mode spectra map of sample LD 0, labelled as Map1 in FTIR data.xlsx. (b) Transimitted light mode line map of LD 0 labelled as Map2 in FTIR data.xlsx. (c) Reflectance mode map of sample LD 0, that uses the same wafer location in (a). Spectra obtained for wafer thickness in FTIR wafter thickness.xlsx. (d) Reflectance mode map of sample LD 0, that uses the same wafter location in (b). Spectra obtained for wafter thickness in FTIR wafter thickness.xlsx.

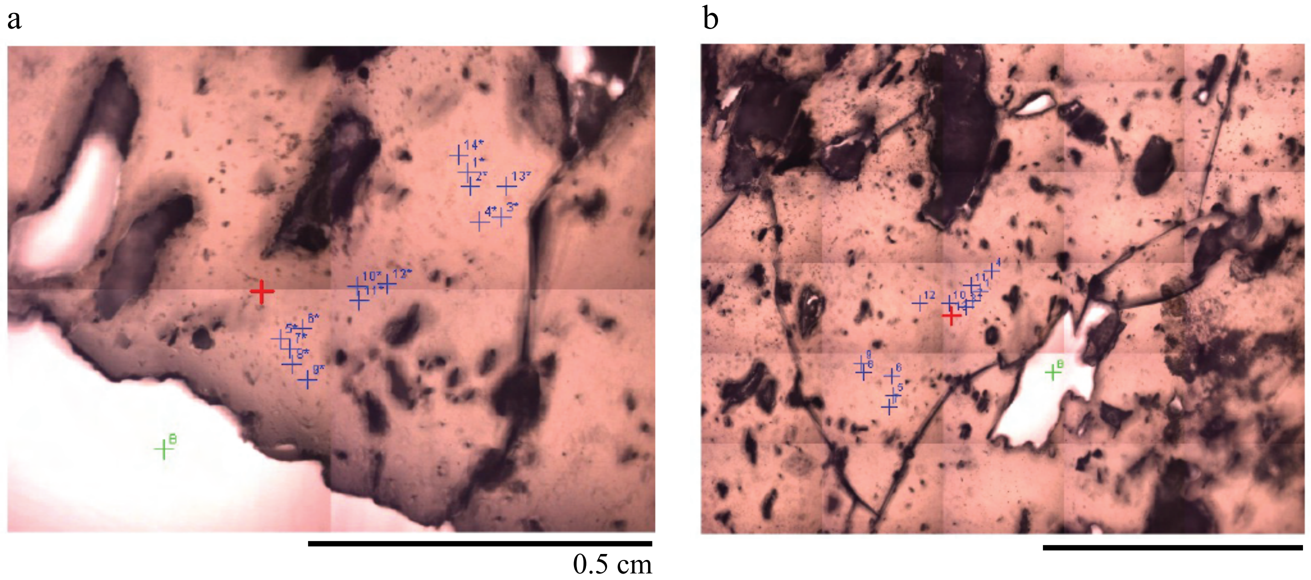

Supplementary Figure 30: (a) Transimitted light mode spectra map of sample LD 6, labelled as Map1 in FTIR data.xlsx. (b) Transimitted light mode line map of LD 6 labelled as Map2 in FTIR data.xlsx.

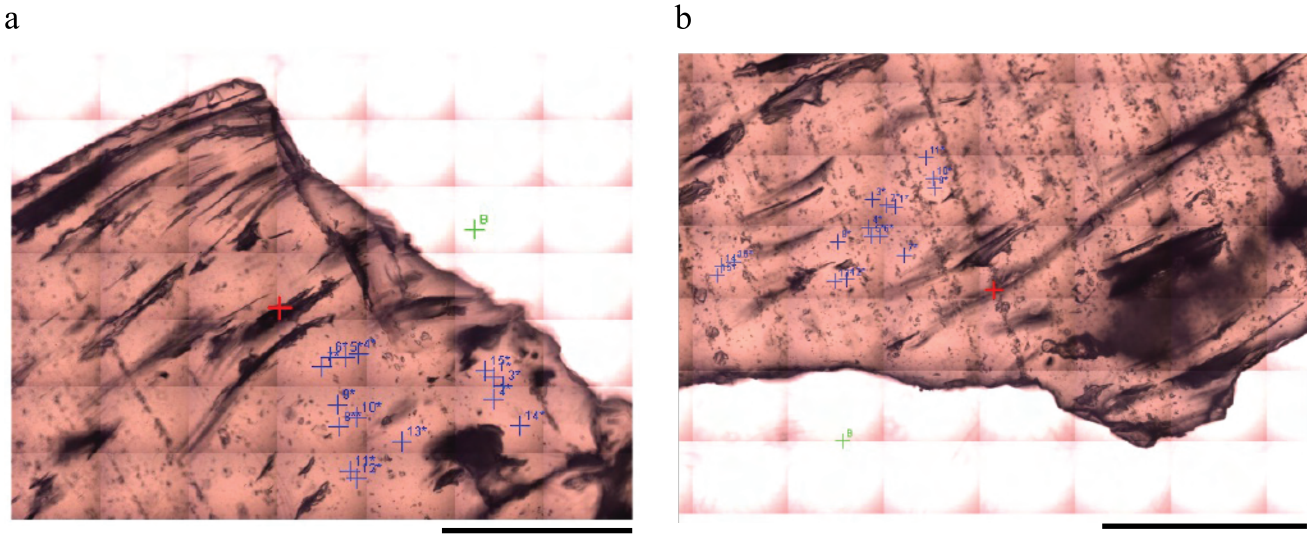

Supplementary Figure 31: (a) Transimitted light mode spectra map of sample LD 21, labelled as Map1 in FTIR data.xlsx. (b) Transimitted light mode line map of LD 21 labelled as Map2 in FTIR

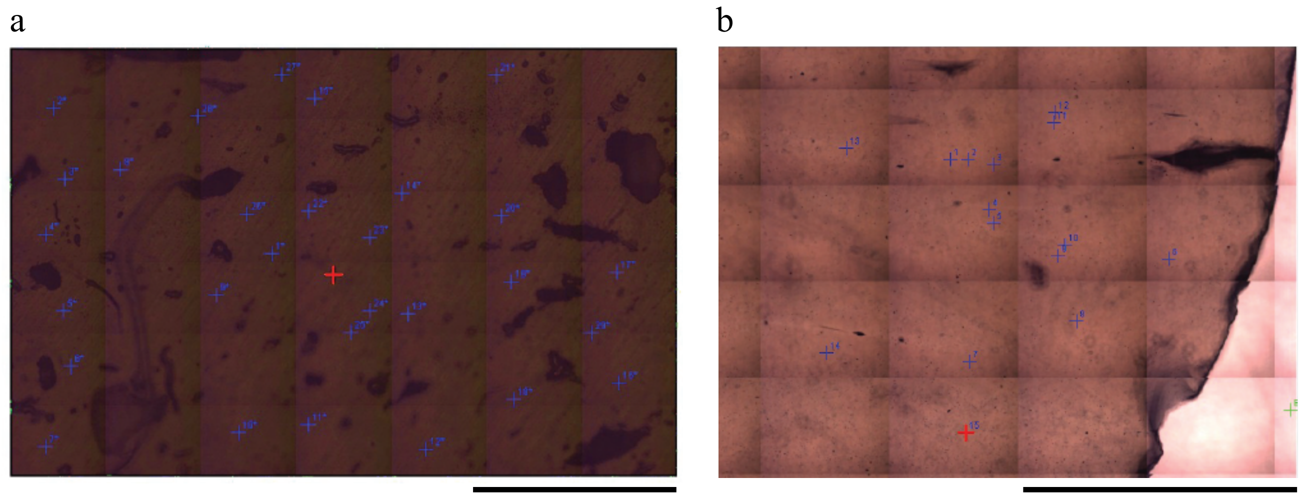

Supplementary Figure 32: (a) Transimitted light mode spectra map of sample LD 31, labelled as Map2 in FTIR data.xlsx. (b) Transimitted light mode line map of LD 31 labelled as Map1 in FTIR data.xlsx.

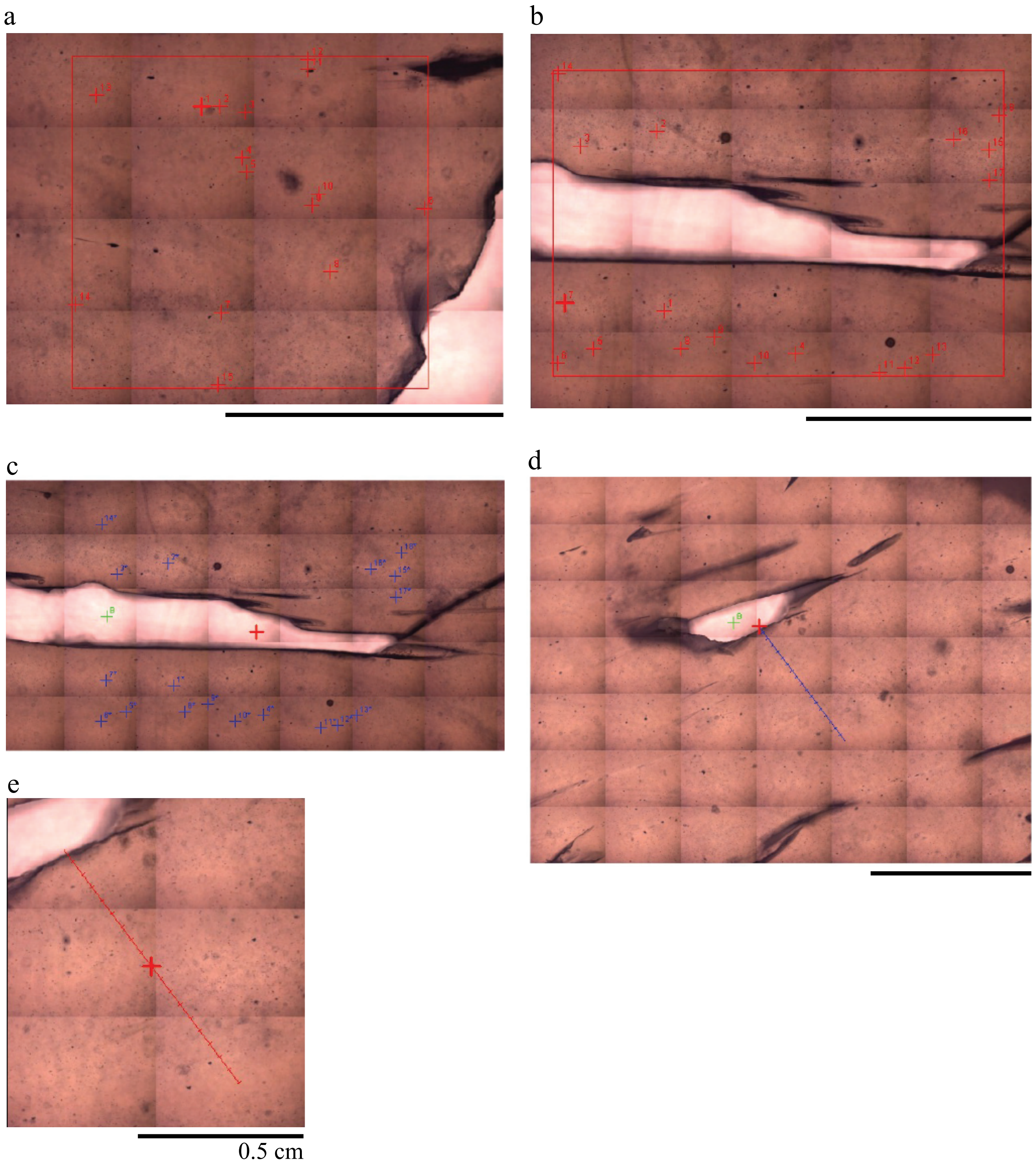

Supplementary Figure 33: (a) Reflectance mode map of sample LD 45. Spectra obtained for wafer thickness in FTIR wafter thickness.xlsx. (b) Reflectance mode map of sample LD 45 that uses the same wafer location in (c). Spectra obtained for wafer thickness in FTIR wafter thickness.xlsx. (c) Transimitted light mode spectra map of sample LD 45, labelled as Map2 in FTIR data.xlsx. (d) Transimitted light mode line map of LD 45 labelled as Line1 in FTIR data.xlsx. (e) Reflectance mode line map of sample LD 45 that uses the same wafer location in (c). Spectra obtained for wafer thickness in FTIR wafter thickness.xlsx.

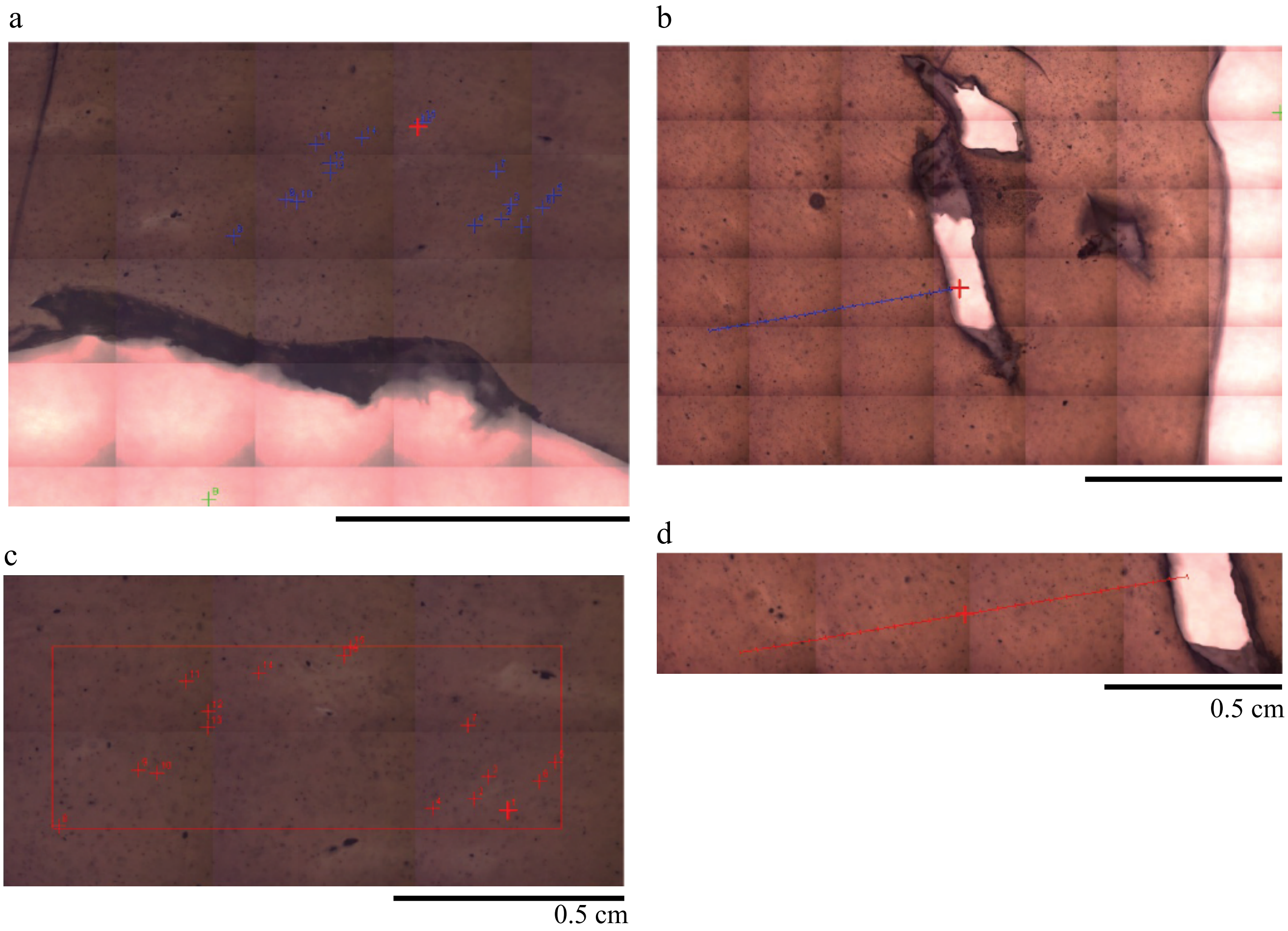

Supplementary Figure 34: (a) Transimitted light mode spectra map of sample LD 76, labelled as Map2 in FTIR data.xlsx. (b) Transimitted light mode spectra map of sample LD 76, labelled as Line1 in FTIR data.xlsx (c) Reflectance mode map of sample LD 76, that uses the same wafter location in (a). Spectra obtained for wafter thickness in FTIR wafter thickness.xlsx. (d) Reflectance mode line map of sample LD 76, that uses the same wafter location in (b). Spectra obtained for wafter thickness in FTIR wafter thickness.xlsx.

LD 84 cm - no recorded spectra or reflected light mode map

a b

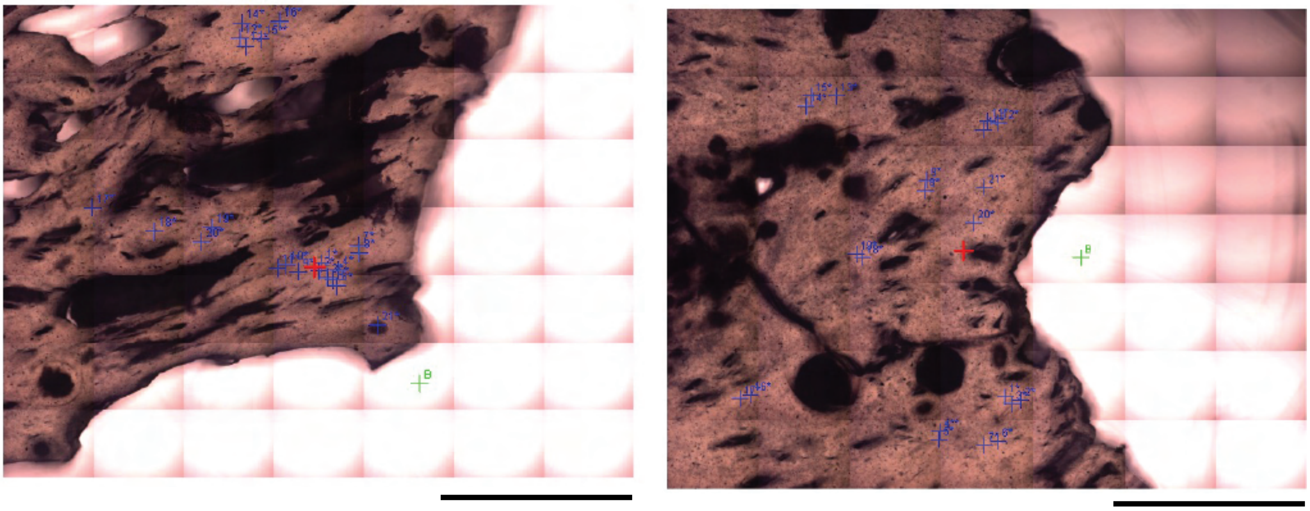

Supplementary Figure 35: (a) Transimitted light mode spectra map of sample LD 92, labelled as Map1 in FTIR data.xlsx. (b) Transimitted light mode line map of LD 92 labelled as Map2 in FTIR data.xlsx.

a b

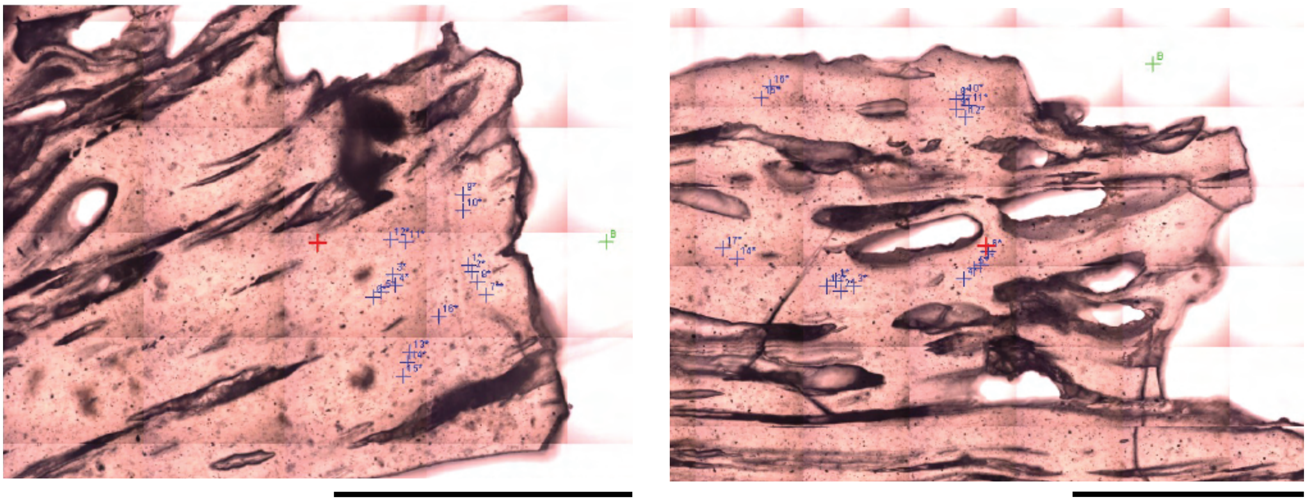

Supplementary Figure 36: (a) Transimitted light mode spectra map of sample LD 93, labelled as Map1 in FTIR data.xlsx. (b) Transimitted light mode line map of LD 93 labelled as Map2 in FTIR data.xlsx.

LD 99 cm - no recorded spectra or reflected light mode map

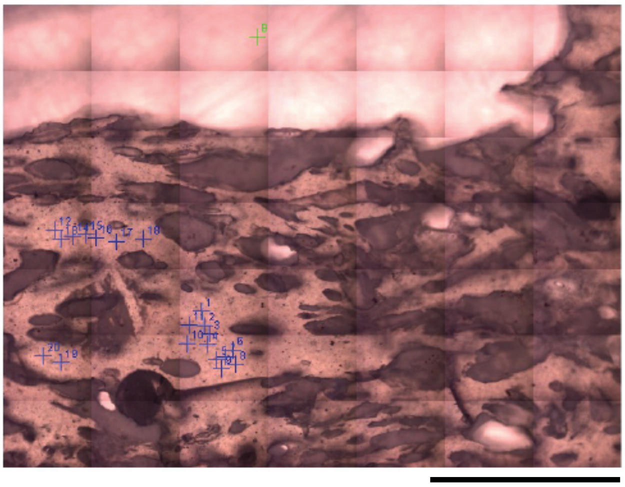

Supplementary Figure 37: Transimitted light mode spectra map of sample LD 100, labelled as Map1 in FTIR data.xlsx.

a b

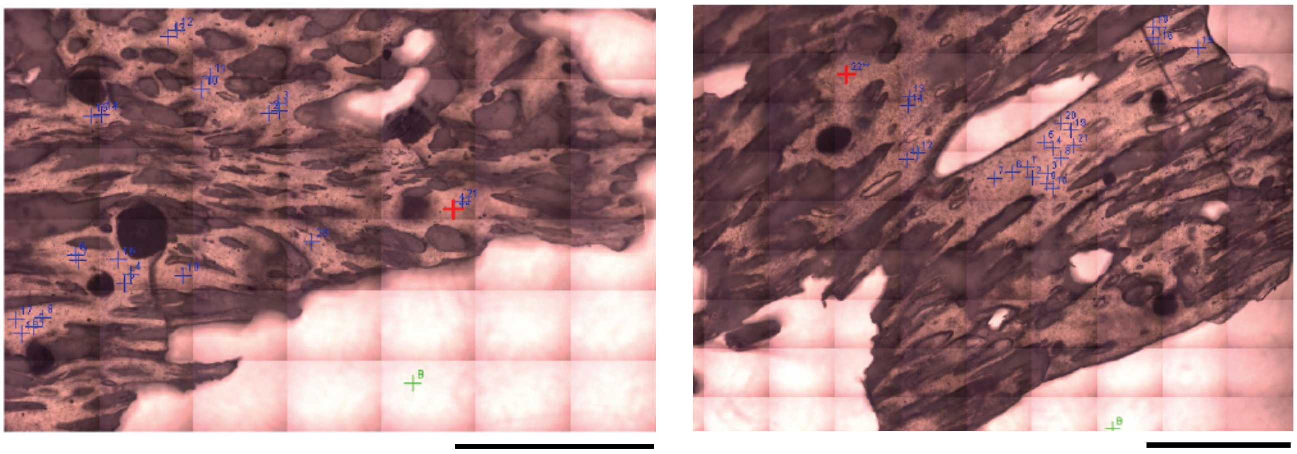

Supplementary Figure 38: (a) Transimitted light mode spectra map of sample LD 101, labelled as Map1 in FTIR data.xlsx. (b) Transimitted light mode line map of LD 101 labelled as Map2 in FTIR

a b

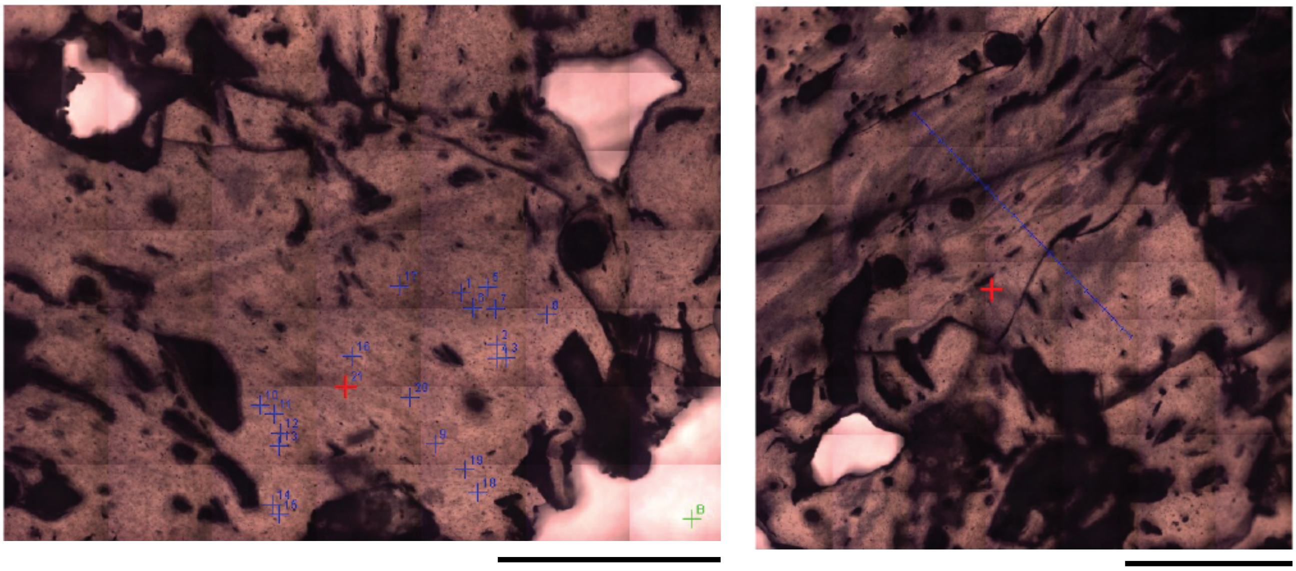

Supplementary Figure 39: (a) Transimitted light mode spectra map of sample LD 110, labelled as Map1 in FTIR data.xlsx. (b) Transimitted light mode line map of LD 110 labelled as Line1 in FTIR data.xlsx.

a b

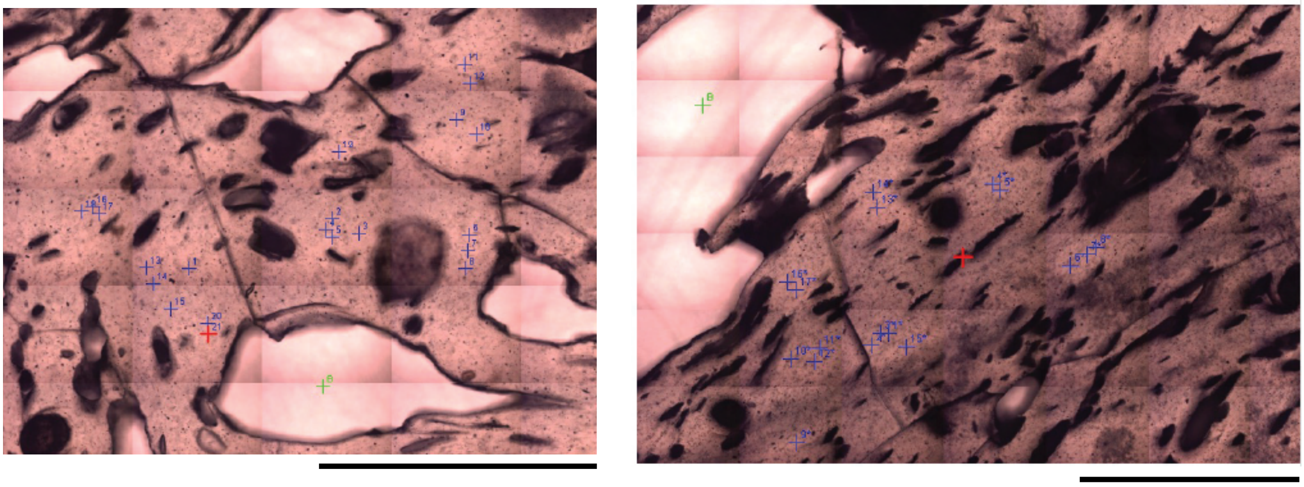

Supplementary Figure 40: (a) Transimitted light mode spectra map of sample LD 117, labelled as Map1 in FTIR data.xlsx. (b) Transimitted light mode line map of LD 117 labelled as Map2 in FTIR data.xlsx.

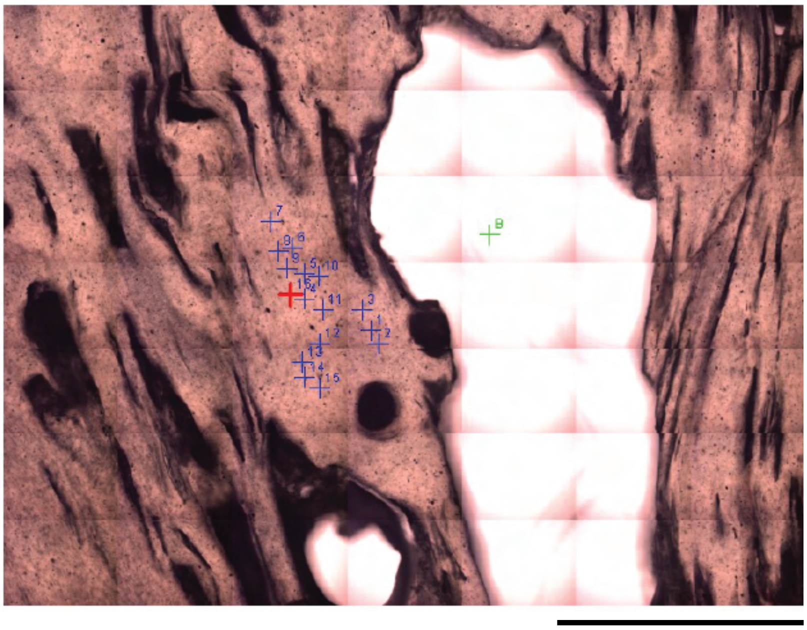

Supplementary Figure 41: Transimitted light mode spectra map of sample LD 118, labelled as Map2 in FTIR data.xlsx.

a b

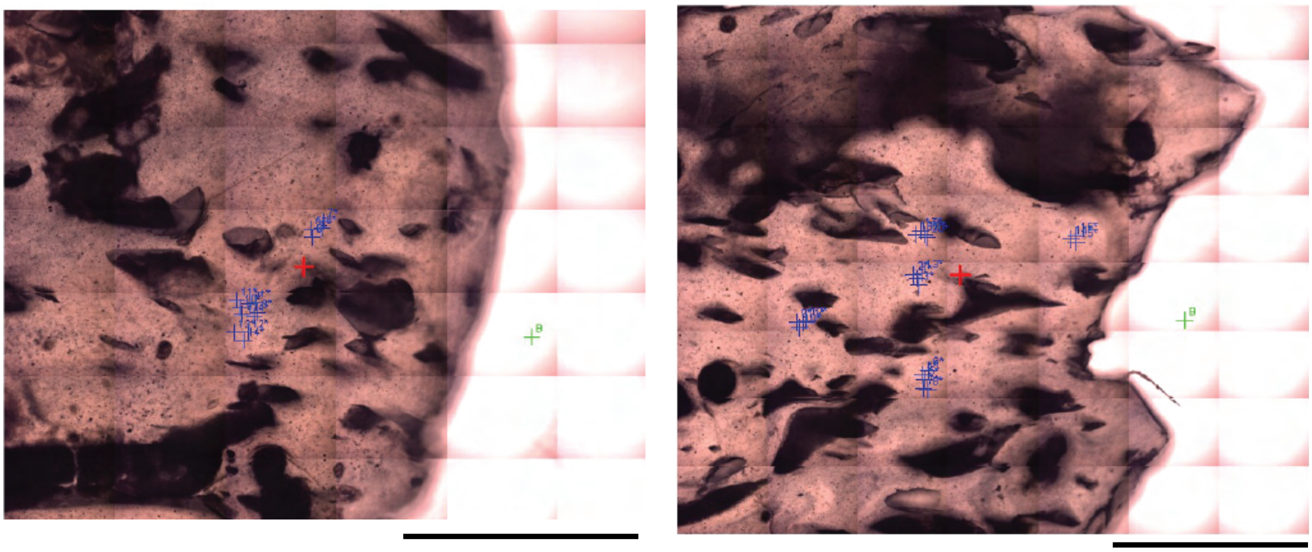

Supplementary Figure 42: (a) Transimitted light mode spectra map of sample LD 125, labelled as Map1 in FTIR data.xlsx. (b) Transimitted light mode line map of LD 125 labelled as Map2 in FTIR data.xlsx.

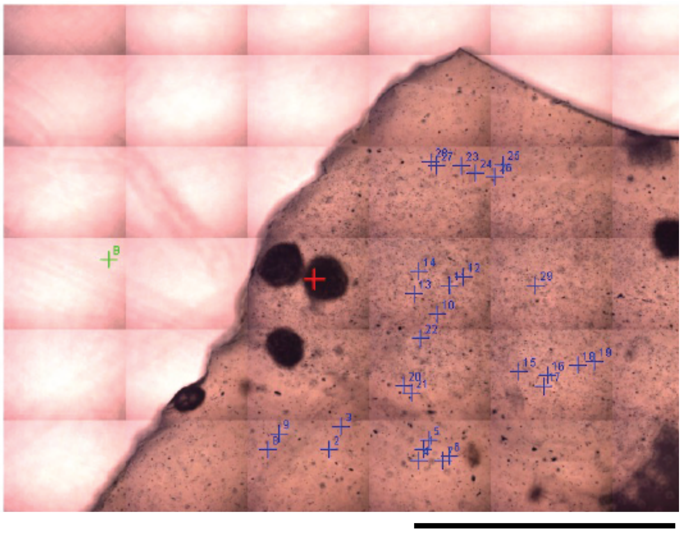

Supplementary Figure 43: Transimitted light mode spectra map of sample LD 140, labelled as Map1 in FTIR data.xlsx.

LD 140 cm - no recorded spectra for Map2 or reflected light mode map

a

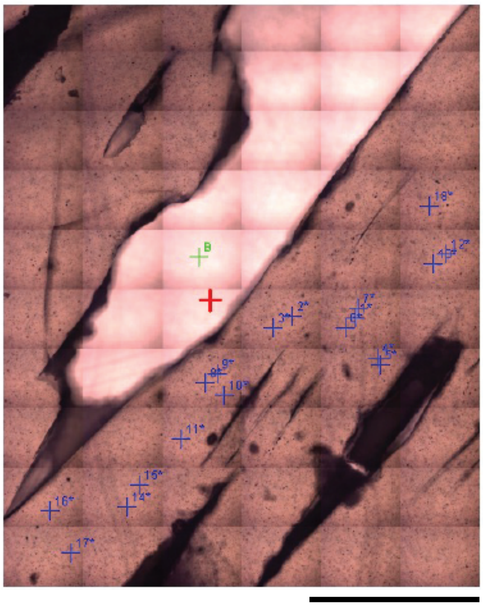

b

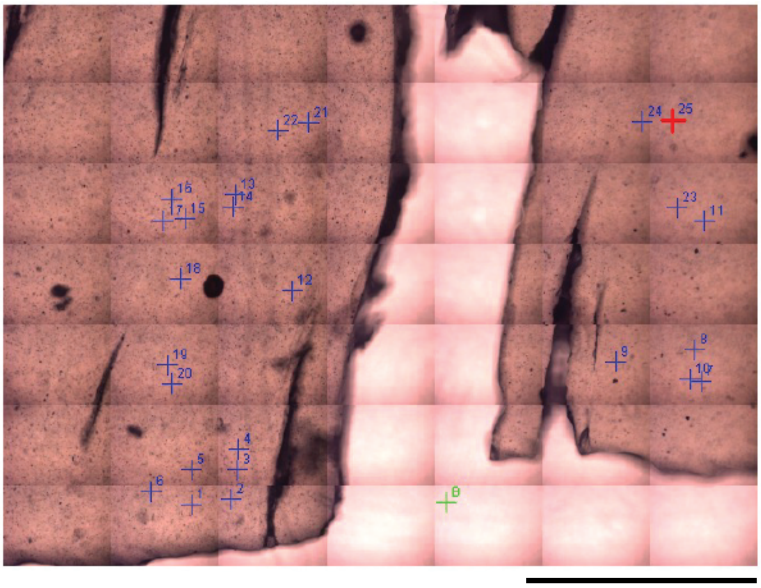

c

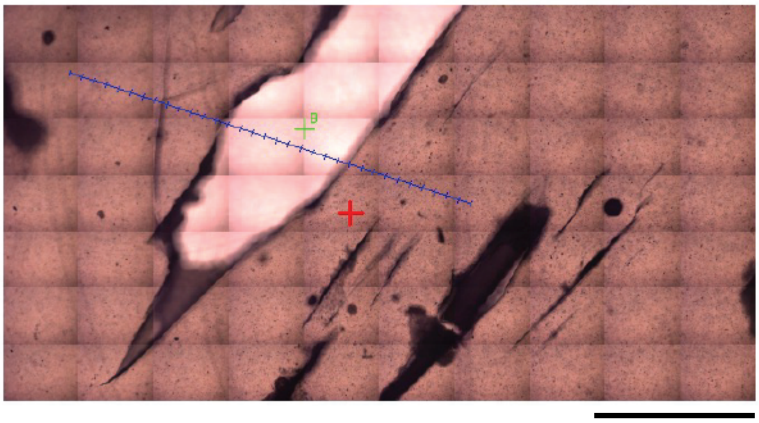

Supplementary Figure 44: (a) Transimitted light mode spectra map of sample LD 168, labelled as Map1 in FTIR data.xlsx. (b) Transimitted light mode line map of LD 168 labelled as Map2 in FTIR data.xlsx. (c) Transimitted light mode line map of LD 168 labelled as Line1 in FTIR data.xlsx.

LD 193 cm - no recorded spectra map or reflected light mode map

a

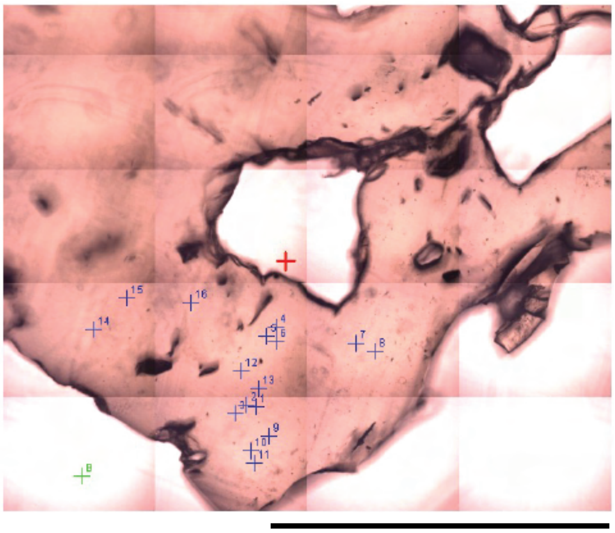

b

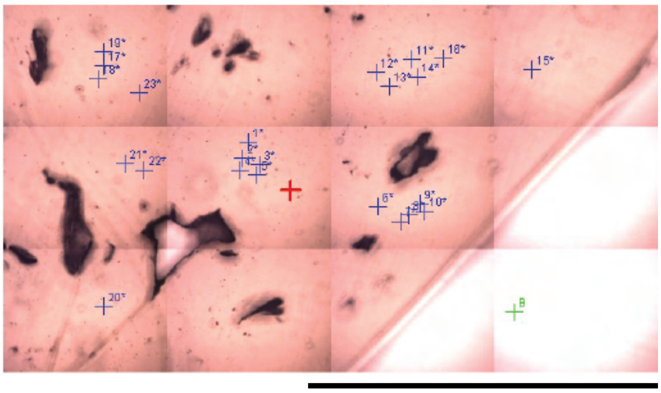

Supplementary Figure 45: (a) Transimitted light mode spectra map of sample LD 222, labelled as Map1 in FTIR data.xlsx. (b) Transimitted light mode line map of LD 222 labelled as Map2 in FTIR data.xlsx.

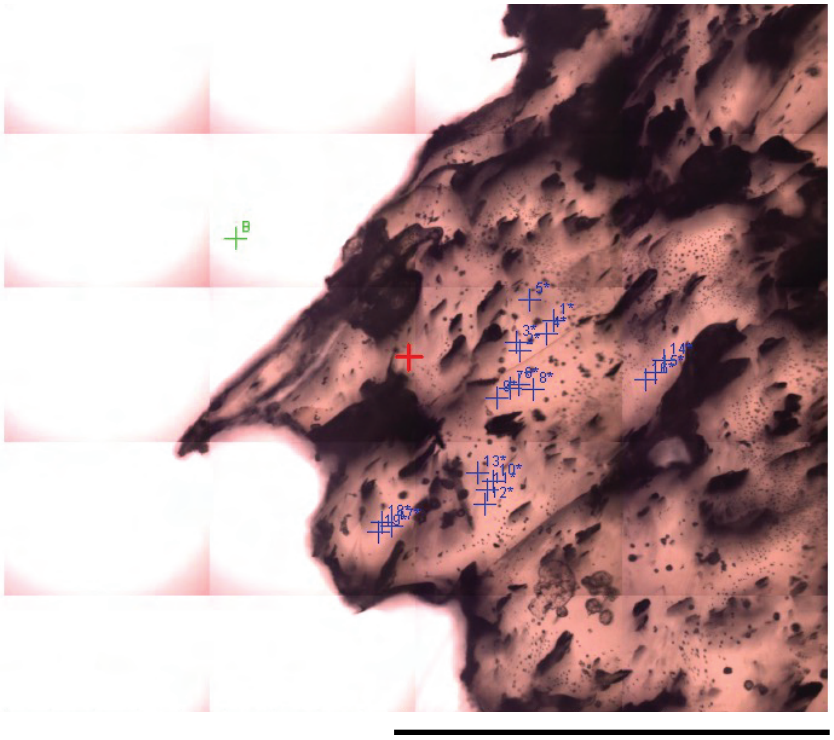

Supplementary Figure 46: Transimitted light mode spectra map of sample LD 272, labelled as Map2 in FTIR data.xlsx.

Upper dyke (UD) 1 cm - no recorded spectra map or reflected light mode map

a b

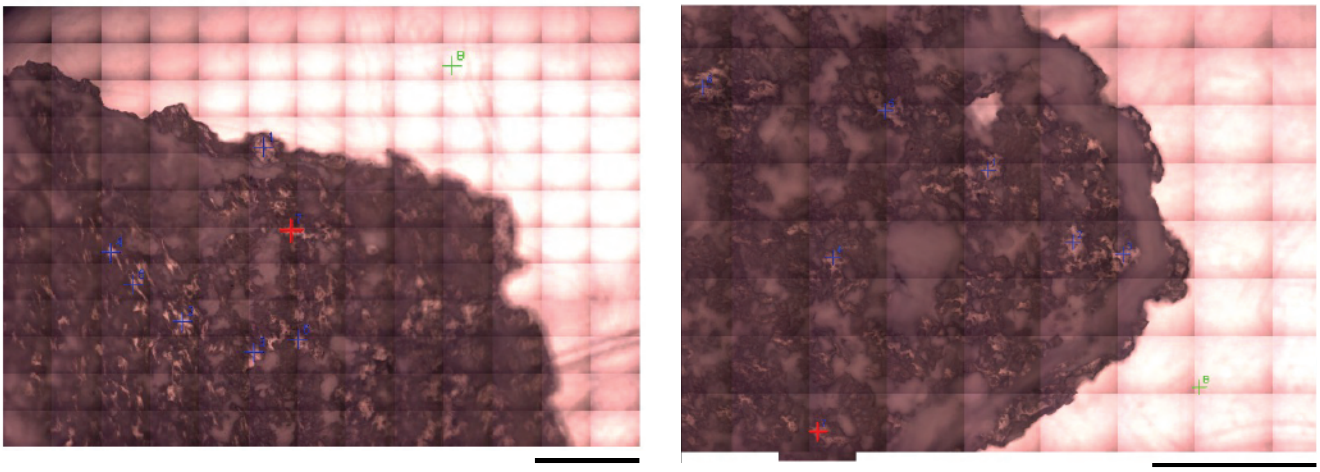

Supplementary Figure 47: (a) Transimitted light mode spectra map of sample 13 from the upper dyke (UD) outcrop labelled as Map1 in FTIR data.xlsx. (b) Transimitted light mode line map of UD 13 labelled as Map2 in FTIR data.xlsx.

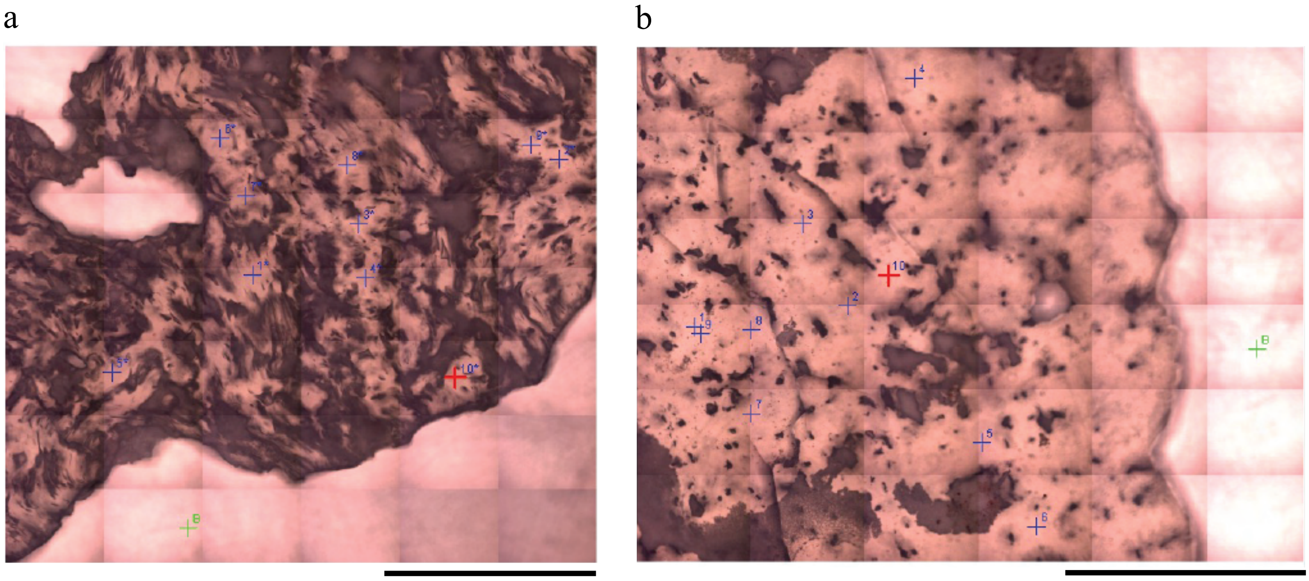

Supplementary Figure 48: (a) Transimitted light mode spectra map of sample UD 15, labelled as Map1 in FTIR data.xlsx. The blue spots were re-run in reflected light mode so thickness measurements are taken from these spots too. (b) Transimitted light mode spectra map of sample UD 15, labelled as Map2 in FTIR data.xlsx. The blue spots were re-run in reflected light mode so thickness measurements are taken from these spots too.

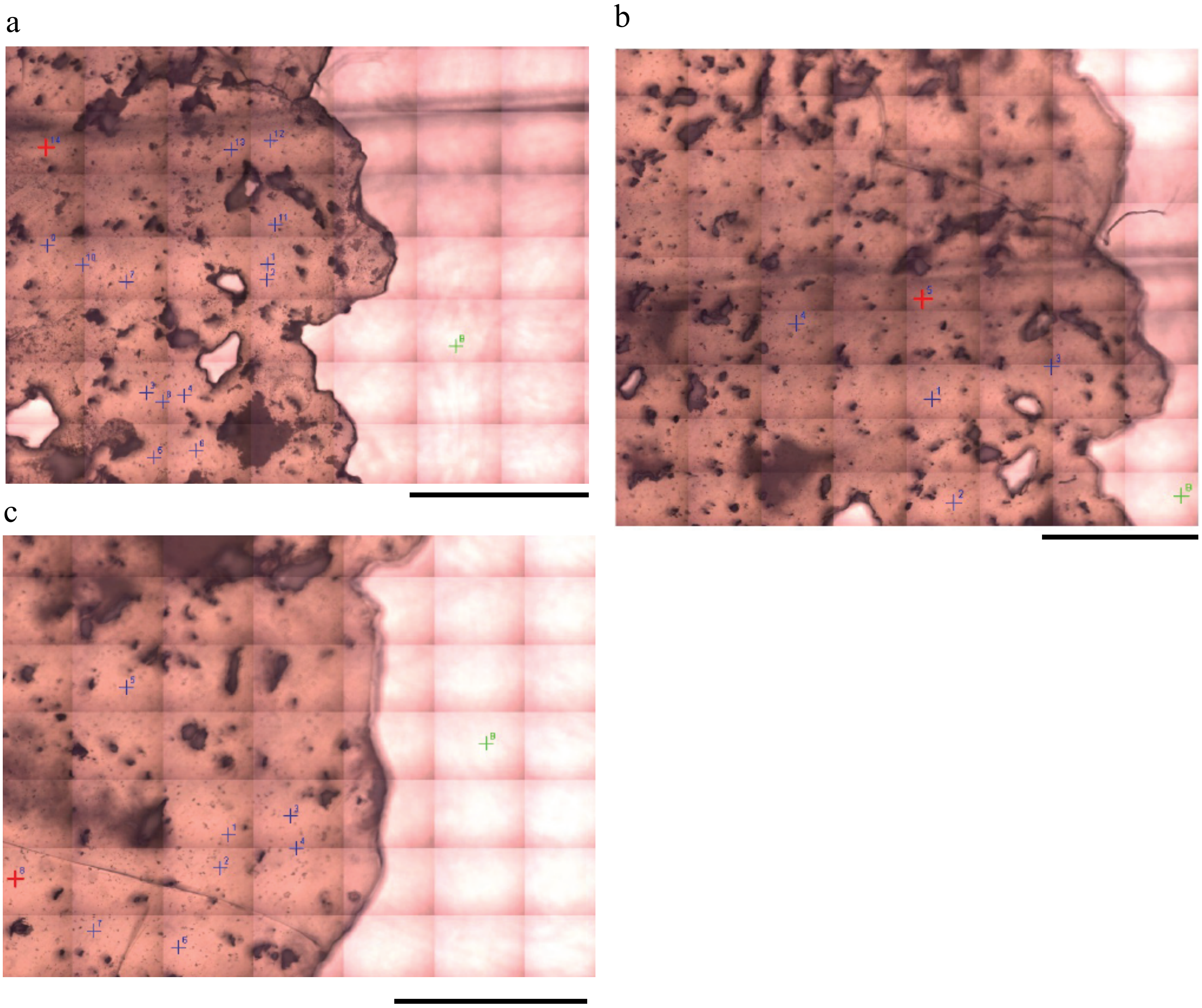

Supplementary Figure 49: (a) Transimitted light mode spectra map of sample UD 17, labelled as Map1 in FTIR data.xlsx. (b) Reflectance mode map of sample UD 17, that uses the same wafter location in (a). Spectra obtained for wafter thickness in FTIR wafter thickness.xlsx. (c) Transimitted light mode spectra map of sample UD 17, labelled as Map2 in FTIR data.xlsx. The blue spots were re-run in reflected light mode so thickness measurements are taken from these spots too.

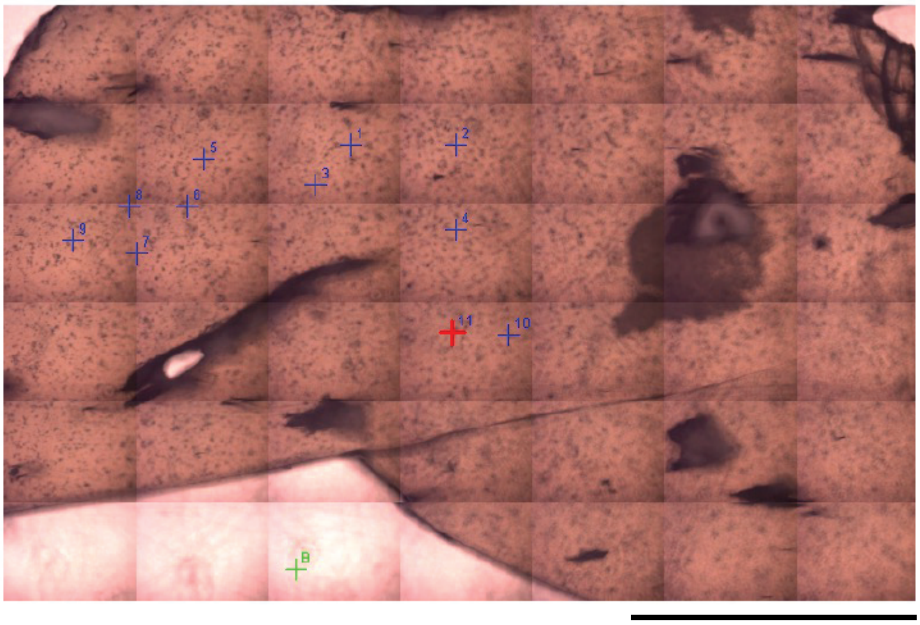

Supplementary Figure 50: Transimitted light mode spectra map of sample UD 20, labelled as Map1 in FTIR data.xlsx. The blue spots were re-run in reflected light mode so thickness measurements are taken from these spots too.

UD 20 cm - no recorded spectra map 2 or reflected light mode map 2

a b

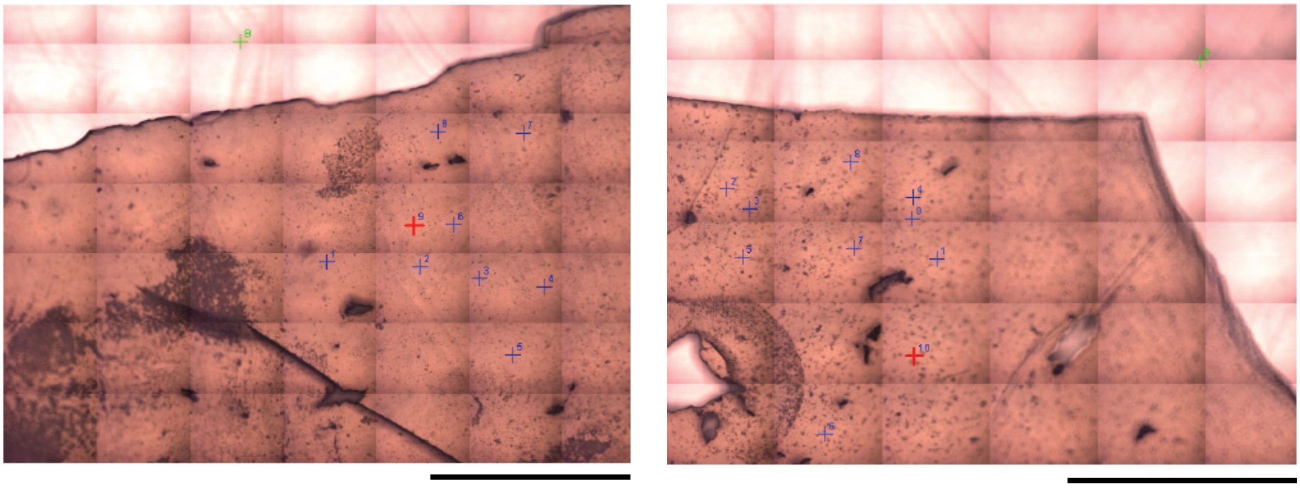

Supplementary Figure 51: (a) Transimitted light mode spectra map of sample UD 30, labelled as Map1 in FTIR data.xlsx. The blue spots were re-run in reflected light mode so thickness measurements are taken from these spots too. (b) Transimitted light mode spectra map of sample UD 30, labelled as Map2 in FTIR data.xlsx. The blue spots were re-run in reflected light mode so thickness measurements are taken from these spots too.

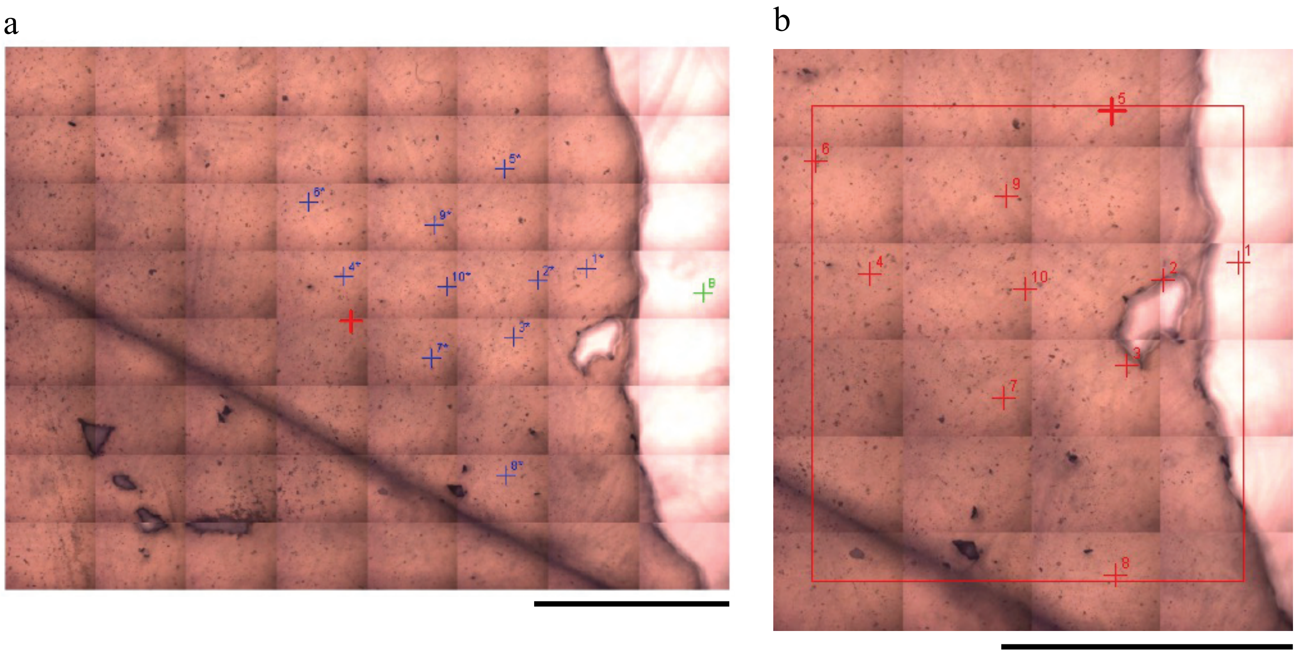

Supplementary Figure 52: (a) Transimitted light mode spectra map of sample UD 32 labelled as Map1 in FTIR data.xlsx. (b) Reflectance mode map of sample UD 32, that uses the same wafter location in (a). Spectra obtained for wafter thickness in FTIR wafter thickness.xlsx.

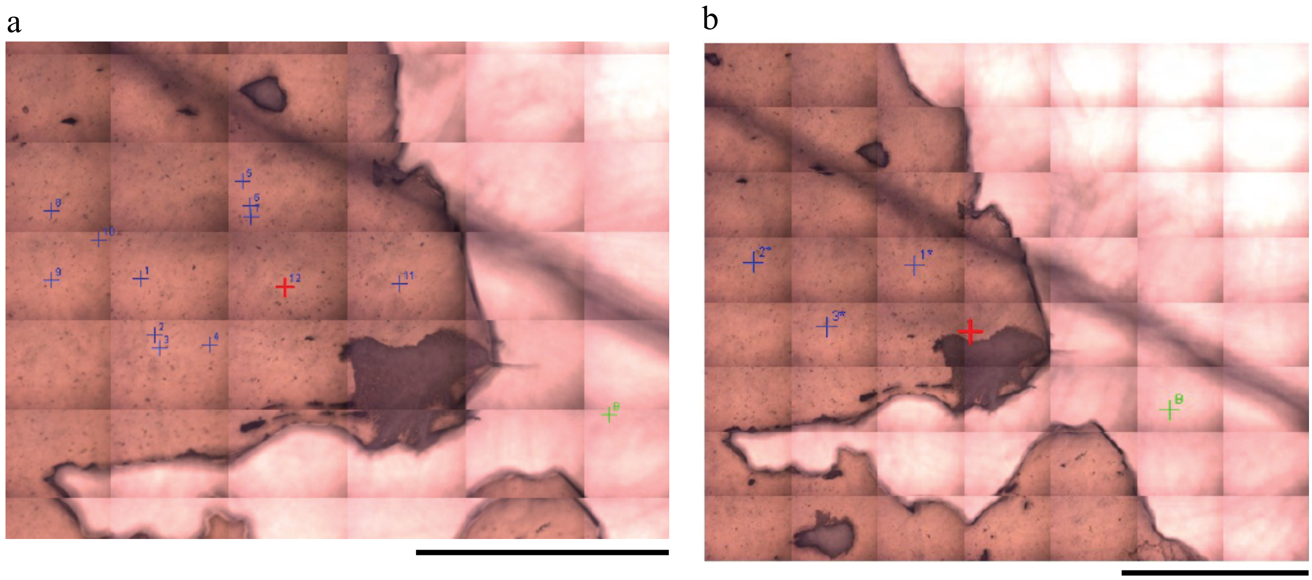

Supplementary Figure 53: (a) Transimitted light mode spectra map of sample UD 35 labelled as Map1 in FTIR data.xlsx. (b) Reflectance mode map of sample UD 35, that uses the same wafter location in (a). Spectra obtained for wafter thickness in FTIR wafter thickness.xlsx.

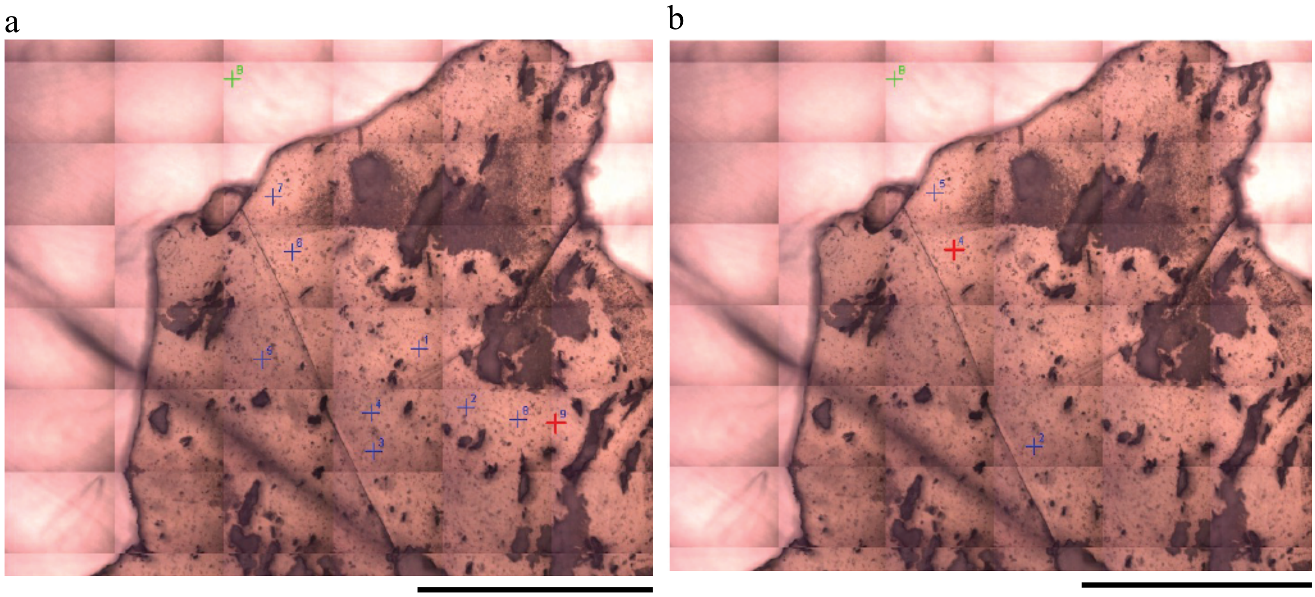

Supplementary Figure 54: (a) Transimitted light mode spectra map of sample UD 35 labelled as Map2 in FTIR data.xlsx. (b) Reflectance mode map of sample UD 35, that uses the same wafter location in (a). Spectra obtained for wafter thickness in FTIR wafter thickness.xlsx.

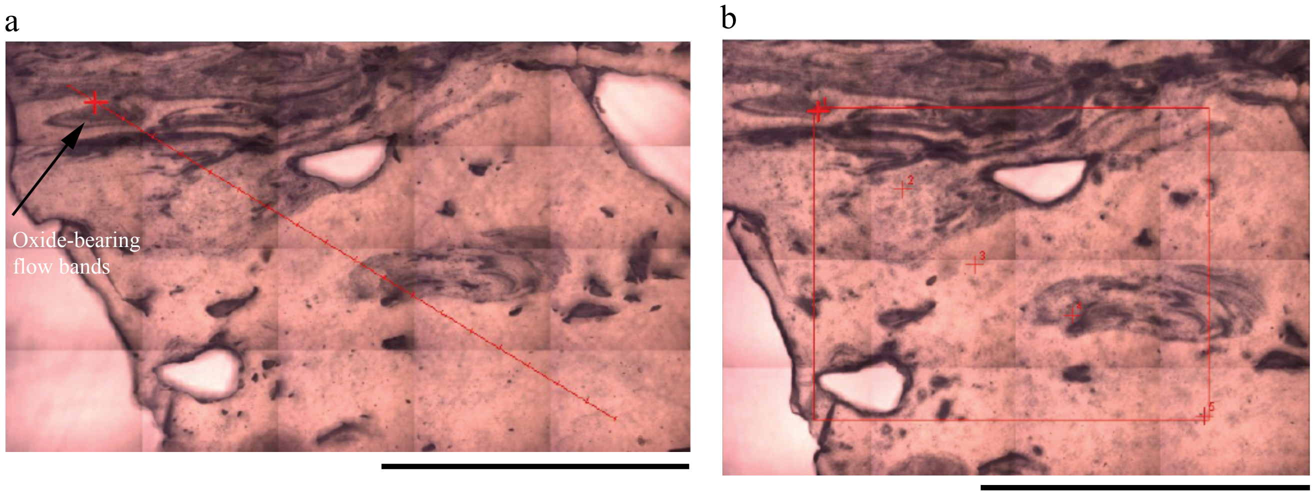

Supplementary Figure 55: (a) Transimitted light mode spectra map of sample UD 50 labelled as Line1 in FTIR data.xlsx. (b) Reflectance mode map of sample UD 50, that uses the same wafter location in (a). Spectra obtained for wafter thickness in FTIR wafter thickness.xlsx.

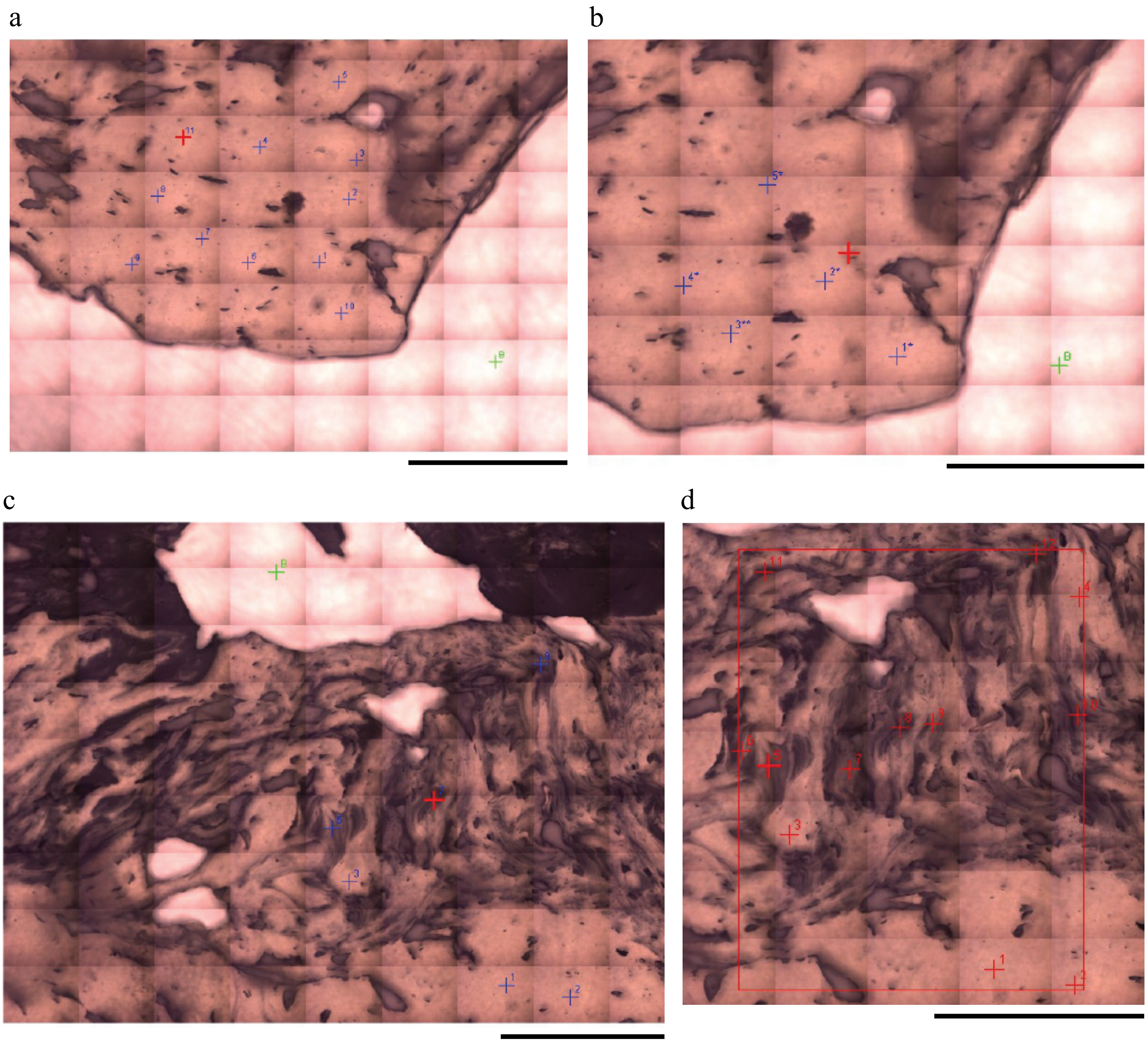

Supplementary Figure 56: (a) Transimitted light mode spectra map of sample UD 50 labelled as Map1 in FTIR data.xlsx. (b) Reflectance mode map of sample UD 50, that uses the same wafter location in (a). Spectra obtained for wafter thickness in FTIR wafter thickness.xlsx. (c) Transimitted light mode spectra map of sample UD 50 labelled as Map2 in FTIR data.xlsx. (d) Reflectance mode map of sample UD 50, that uses the same wafter location in (c). Spectra obtained for wafter thickness in FTIR wafter thickness.xlsx.
