# Supplementary material for: Evidence for the formation of silicic lava by pyroclast sintering
Source: Nat Commun. 2024 Jun 24;15:5347. doi: 10.1038/s41467-024-49601-6 (PMC11196653; doi:10.1038/s41467-024-49601-6)
Supplement: Supplementary file 3 — Description of Additional Supplementary Files [file 41467_2024_49601_MOESM3_ESM.pdf]

Description of Additional Supplementary Files

Supplementary Data 1
This dataset contains the information collected from FTIR to calculate water concentration for both feeder dyke samples. The midpoint water calculations were used in Fig. 2m and 2n on this manuscript. It also repeats water data collected from Tuffen and Castro (2009) for convenience.

Tuffen, H. & Castro, J. M. The emplacement of an obsidian dyke through thin ice: Hrafntinnuhryggur, Krafla Iceland. *J. Volcanol. Geotherm. Res.* **185**, 352–366 (2009).

Supplementary Data 2
This dataset contains the wafer thickness measurements of both feeder dyke samples. It contains the thickness measurement calculated through reflective light mode using FTIR, and the thickness calculated from a digital micrometer.
